# Unraveling the Molecular Dance: Insights into TREM2/DAP12 Complex Formation in Alzheimer’s Disease through Molecular Dynamics Simulations

**DOI:** 10.1021/acsomega.4c03060

**Published:** 2024-06-21

**Authors:** Zhiwen Zhong, Martin B. Ulmschneider, Christian D. Lorenz

**Affiliations:** † Biological Physics and Soft Matter Group, Department of Physics, King’s College London, London WC2R 2LS, U.K.; ‡ Department of Chemistry, King’s College London, London SE1 1DB, U.K.

## Abstract

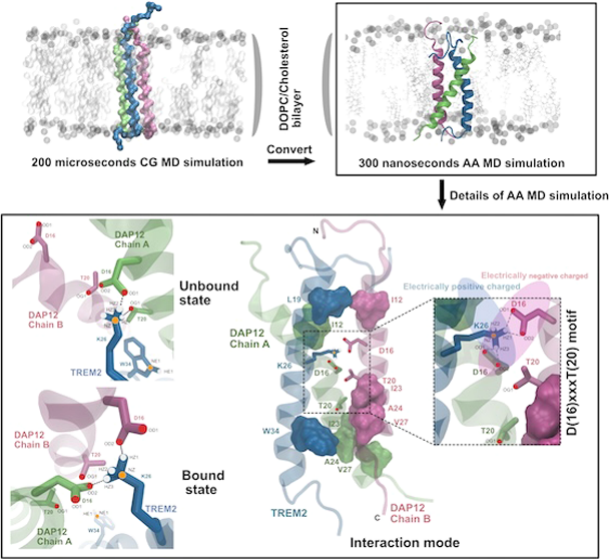

Alzheimer’s disease (AD) is a widespread neurodegenerative condition affecting millions globally. Recent research has implicated variants of the triggering receptor expressed in myeloid cells 2 (TREM2) as risk factors for AD. TREM2, an immunomodulatory receptor on microglial surfaces, plays a pivotal role in regulating microglial activation by association with DNAX-activation protein 12 (DAP12). Despite its significance, the mechanism underlying the formation of the complex between the transmembrane domains (TMDs) of TREM2 and DAP12 remains unclear. This study employs multiscale molecular dynamics (MD) simulations to investigate three TMD complex models, including two derived from experiments and one generated by AlphaFold2. Conducted within a lipid membrane consisting of an 80:20 mixture of phosphatidylcholine (POPC) and cholesterol, our analysis reveals hydrogen-bonding interactions between K26 of TREM2 and D16 of DAP12 in all three models, consistent with previous experimental findings. Our results elucidate the different spatial conformations observed in the models and offer insights into the structure of the TREM2/DAP12 TMD complex. Furthermore, we elucidate the role of charged residues in the assembly structure of the complex within the lipid membrane. These findings enhance our understanding of the molecular mechanism governing TREM2/DAP12 complex formation, providing a foundation for designing novel therapeutic strategies to address AD and other neurodegenerative diseases.

## Introduction

Alzheimer’s disease (AD) is a prevalent neurodegenerative disease that is the leading cause of dementia in the elderly.^1^ It was first identified over a century ago by the German neuropsychiatrist, Alois Alzheimer,^2^ and now has been proven to have a complicated genetic architecture.^3^ The encoding genes, such as presenilin-1 (PSEN1) and presenilin-2 (PSEN2), which are part of the γ-secretase complex, along with the ϵ4 allele of apolipoprotein E (APOE) and amyloid precursor protein (APP), exhibit a high degree of mutation penetrance. Amyloid plaques are one of the hallmarks of AD^4^ and are mainly composed of β-amyloid peptides (Aβs), which are derived from the APP.^5^ The triggering receptor expressed on myeloid cells 2 (TREM2) is a transmembrane protein that is thought to be related to AD in two significant ways. First, whole-genome analyses have identified rare variants in TREM2 that increase the risk of AD by approximately 3-fold,^6,7^ similar to APOE. Second, TREM2 is selectively expressed in myeloid cells, where it promotes the optimal microglial function required to attenuate AD progression. Microglia, which are the primary component of the human brain’s immune system, are involved in Aβ accumulation and thus contribute to the development of AD.^8^ Thus, TREM2 is a potential target for eliciting a protective role for microglia in AD and other neurodegenerative diseases. DAP12 (DNAX-activating protein of 12 kDa) is a transmembrane protein that activates downstream signaling pathways by binding to TREM2.^9,10^ Together, TREM2 and DAP12 play crucial roles in the regulation of microglial function, including phagocytosis and inflammatory responses.

The initiation of the TREM2 signaling pathway relies on a stable interaction between TREM2 and DAP12 within the membrane. Researchers proposed that TREM2 could bind to numerous molecules, and its signaling intensity and direction could be differentially influenced by interacting with various ligands.^11^ The TREM2 pathway is first triggered when the N-terminus of TREM2 binds to ligands in the extracellular domain, which activates downstream pathways that involve a stable interaction between TREM2 and DAP10/12.^12^ Then, the ectodomain is cleaved off by metalloproteinase domain-containing proteins 10/17 (ADAM 10/17), and the C-terminal fragment (CTF) is cleaved by γ-secretase (a γ-secretase high-resolution cryo-EM structure has recently been solved^13^), ultimately terminating the TREM2 pathway.^14,15^ The termination of the TREM2 signaling pathway allows microglia to return to their basal state. Mutations in TREM2 and DAP12 genes have been linked to an increased risk of developing neurodegenerative diseases such as Alzheimer’s disease.^16^ Understanding the molecular mechanisms underlying the TREM2 signaling pathway and associated signaling adaptor molecules such as DAP12 is critical for the development of new therapeutic strategies for neurodegenerative diseases.^17,18^ Further research on this pathway will provide valuable insights into the regulation of microglial function and the potential development of new drugs targeting neurodegenerative diseases.

Recent studies have revealed that TREM2 binds to the DAP12 dimer via salt bridges that involve an aspartic acid on each chain of the DAP12 protein and lysine in TREM2 and, therefore, ultimately forms a trimer.^19^ The orientation of the transmembrane domain of the TREM2 monomer and the DAP12 dimer extends from the N-terminus to the C-terminus, spanning from the outer cell membrane to the inner cell membrane. Furthermore, researchers have demonstrated that the TREM2 transmembrane helix is stabilized by either binding to DAP12 or as a result of the mutation of the charged amino acid K26 to an alanine (K26A).^20^ The presence of a charged amino acid (K26) in the transmembrane domain leads to the adoption of a kinked structure by the TREM2 transmembrane domain (TMD), which exhibits greater flexibility. Conversely, the removal of this charge (K26A) results in the stabilization of the TREM2-TMD, thereby reducing its dynamics and resulting in its structure being similar to when it is complexed with DAP12.^20^ While there is substantial interest in understanding how TREM2 recognizes DAP12 in the membrane, a comprehensive understanding of the atomistic interactions governing the formation of this stable complex is still lacking. Molecular dynamics (MD) simulations offer a distinct opportunity to foster this level of understanding. By modeling the interatomic interactions, MD simulations predict the movements of individual atoms in complex molecular systems, providing insights into fundamental biomolecular processes such as protein folding, ligand binding, and conformational changes.^21^ The growing accessibility of experimentally determined structures, exemplified by repositories like the Protein Data Bank (PDB),^22^ the evolution of machine learning techniques for predicting protein structures (such as AlphaFold2),^23^ and the continuous enhancement of computational hardware have significantly bolstered the prominence of MD simulations. These advancements facilitate the simulation of larger and more intricate molecular systems within reduced time frames.

**Figure 1:**
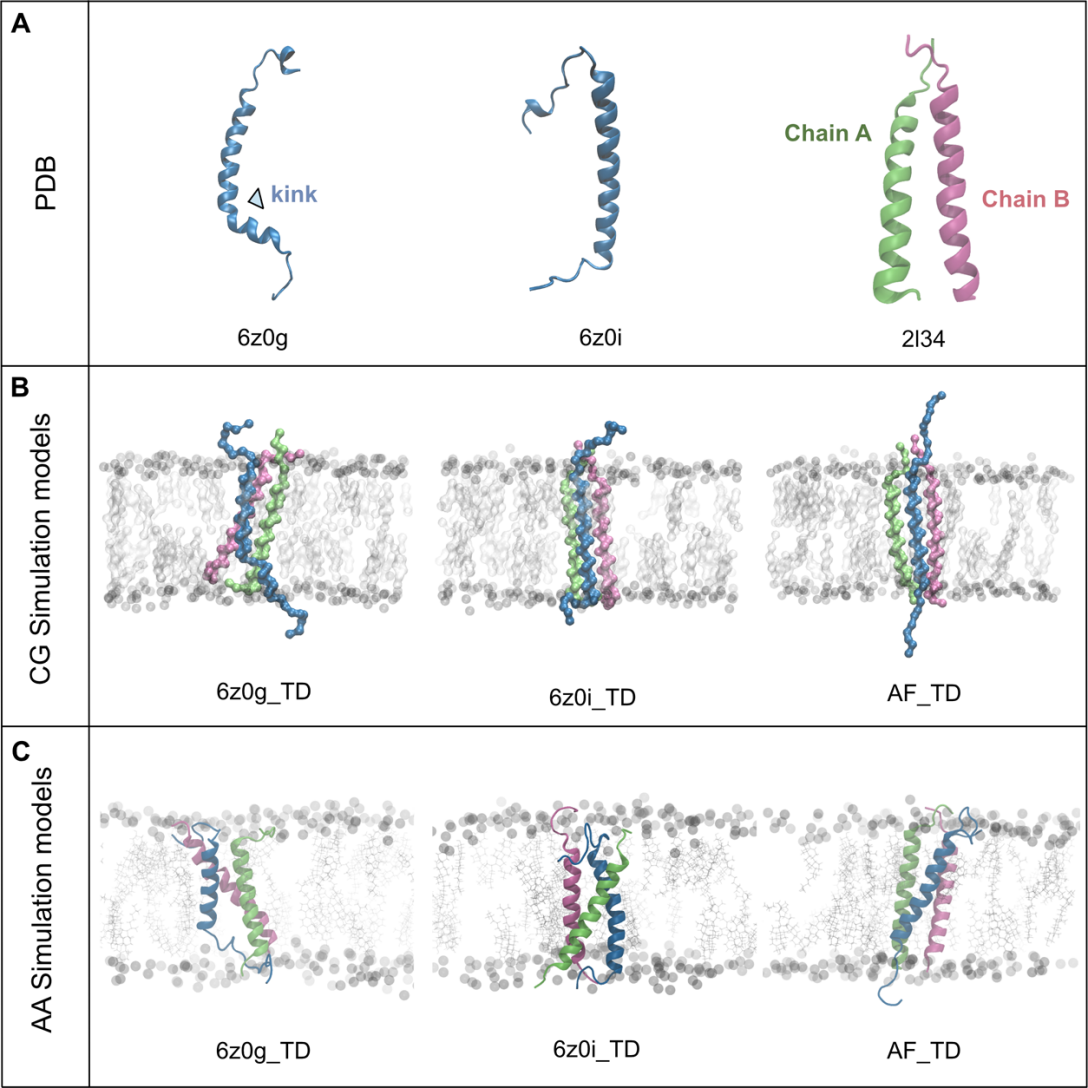
Representation of three TREM2/DAP12 (TD) transmembrane domain complexes in a lipid environment. (A) From left to right: 6z0g illustrates the kinked structure of TREM2, while 6z0i depicts the TREM2 PDB structure interacting with DAP12. The rightmost structure portrays the DAP12 dimer, with Chain A in lime and Chain B in mauve. (B) Illustration of the coarse-grained 3D structure of the three models, where the gray sphere represents the POPC bilayer, and cholesterols are visualized using a licorice representation. Protein structure colors are consistent with (A). (C) All-atom 3D structures of the three models within the POPC/cholesterol environment. The gray sphere indicates the POPC bilayer, and silver dots denote cholesterol.

In this study, we studied three models of the TREM2/DAP12 (TD) complex in a phosphatidylcholine (POPC) and cholesterol (80:20) lipid environment: two based on experimental data and one modeled using AlphaFold2, involving TREM2/DAP12 complexes simulated within a phosphatidylcholine (POPC) and cholesterol (80:20) lipid environment (Figure 1). Employing long-time frames of coarse-grain (CG) MD simulations to stabilize the systems, we subsequently converted them to all-atom (AA) MD simulations. Our atomic-level analysis revealed unique protein–protein interfaces in the three systems, resulting from a combination of salt bridges, hydrogen bonds, and hydrophobic interactions. Notably, all three systems showcased a K26/D16 interaction involving both a salt bridge and hydrogen bonds. This interaction appears to be crucial in the recognition process of the TD complex.

## Results

### Three TREM2/DAP12 Complexes in a Lipid Environment

We conducted a 200 μs CG simulation and then converted the final configuration to an AA representation. Afterward, we ran the AA system for an additional 300 ns within the same lipid environment. The system’s stability after the 200 μs simulations was confirmed by assessing the total number of contacts between the proteins (Figure S1) and the contact maps between individual residues within the various proteins (Figures S2–S4). The contact maps depict consistent contact between residues in the central region of the different proteins across four different time frames of the trajectories.

Of the three systems we studied (Figure 1C), 6z0g represents the NMR structure of apo TREM2, while 6z0i depicts the NMR structure of TREM2 when it is bound with DAP12 (Figure 1A), although only the TREM2 transmembrane helix part has been resolved due to NMR experimental limitations. However, understanding the entire complex structure and its conformational dynamics is crucial for elucidating the TREM2 and DAP12 binding process. A notable difference between 6z0g and 6z0i in the three-dimensional structure is the presence of a kinked region in the TREM2 transmembrane domain. Subsequently, the DAP12 dimer (PDB: 2l34) was assembled separately with the TREM2 monomers (PDB: 6z0g and 6z0i) and formed 6z0g_TD and 6z0i_TD systems, respectively (Figure 1C). The third system, AF_TD, was generated using AlphaFold2 and shares the same sequence as 6z0g_TD and 6z0i_TD. The confidence level of the AF_TD complex is perceptible and is demonstrated in Figure S5.

### Kink Angles in Three Systems Reveal Different Binding Mechanisms

**Figure 2:**
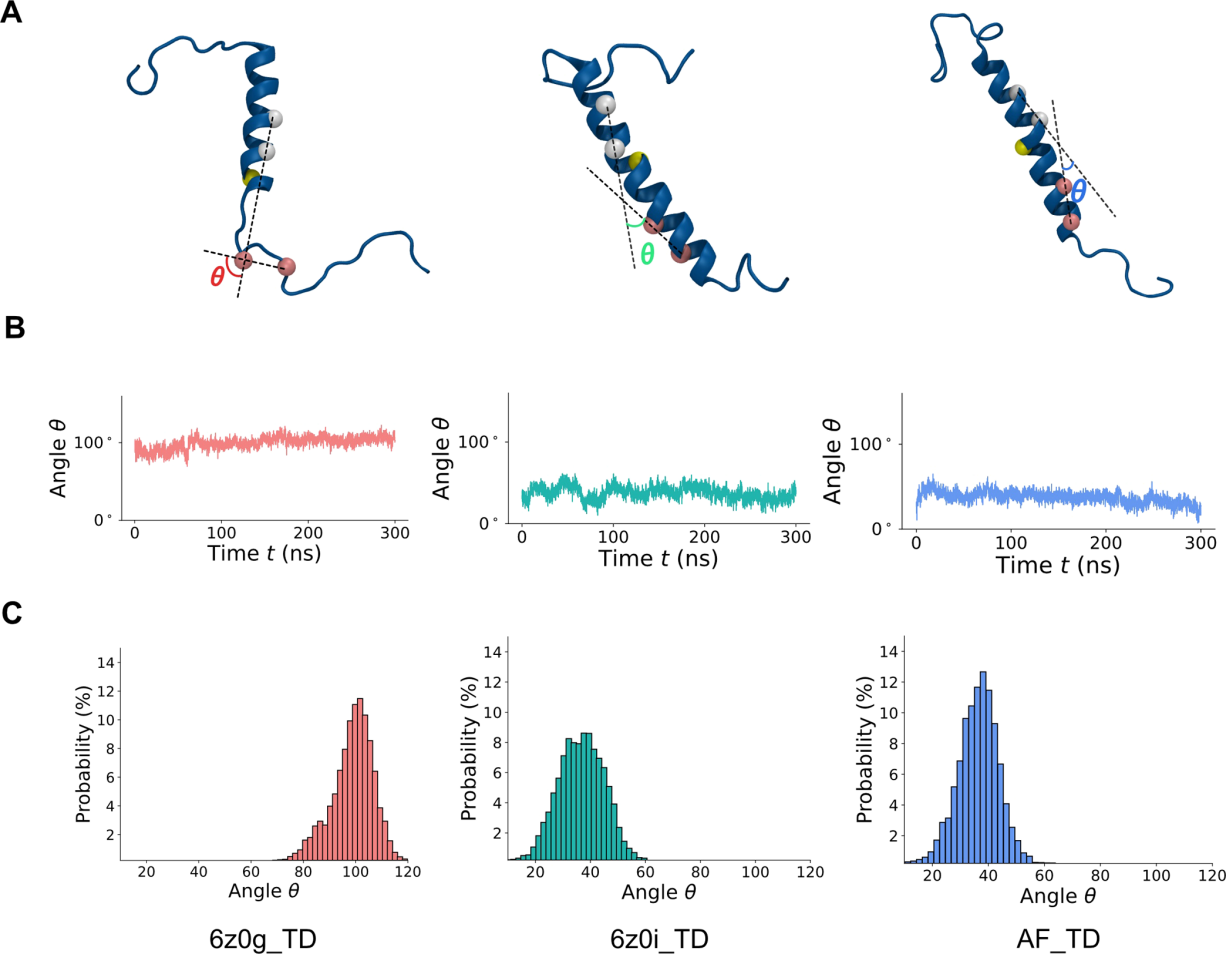
Representation of kink angles in three different systems. (A) Snapshots of TREM2 structures taken at 150 ns. White spheres represent C_α_ atoms of A20 and L24, while pink spheres represent C_α_ atoms of A32 and A36. Yellow spheres represent the C_α_ atom of K26. (B) Diagrams displaying the kink angles of the three models. Red, green, and blue lines correspond to 6z0g_TD, 6z0i_TD, and AF_TD, respectively. (C) Illustrations showcasing the distribution of kink angles. Colors are consistent with (B).

Within all three systems, TREM2 exists as a single α-helix transmembrane protein, while DAP12 is a dimer. Notably, the 6z0g_TD system exhibits a kink, while 6z0i_TD and AF_TD show no kinks, consistent with the apo PDB structures (Figure 1A). We determined the kink angle using four C_α_ atoms in the TREM2 α-helix (shown in Figure 2A). During the 300 ns simulation, the average kink angle for 6z0g_TD measured 99.8 ± 0.1°, whereas 6z0i_TD and AF_TD showed kink angles of 37.7 ± 0.1 and 37.4 ± 0.1°, respectively (Figure 2B). Upon analysis of the kink angle distributions, we observed that the most frequent kink angle for 6z0g_TD is approximately 101 ± 1° (Figure 2C). In contrast, 6z0i_TD and AF_TD displayed less pronounced kinks, with the most prevalent angles measuring 38 ± 1 and 40 ± 1°, respectively. These findings suggest that after our extended simulations (200 μs CG simulation and 300 ns AA simulation), the kink persisted in the 6z0g_TD model, while the other two models exhibited less pronounced kinks. This implies that different TREM2-DAP12 binding modes exist among the three systems.

### Contact Maps Using C_α_ Atoms Distance as Cutoff Reveal the Interaction between TREM2 and Two Chains of DAP12

**Figure 3:**
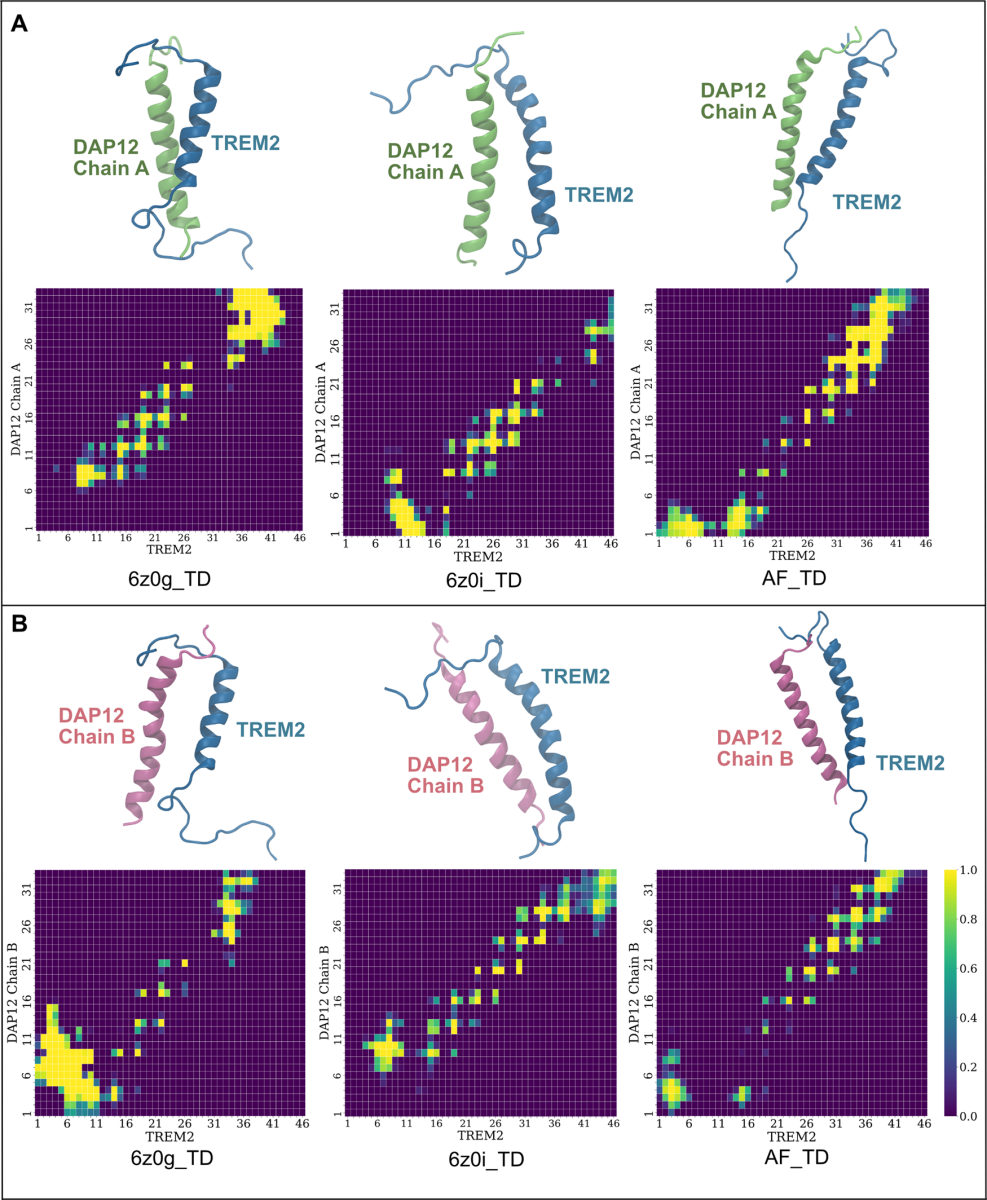
Snapshots of the 3D structures and contact maps of the three systems. The snapshot was taken at 200 ns, and the contact map was generated between 175 and 225 ns. Part (A) illustrates TREM2 contact with DAP12 Chain A in the three models. DAP12 Chain A structures are depicted in lime, while TREM2 is shown in blue. The contact map utilizes the “rocket” preset color scheme. Part (B) showcases TREM2 contacts with DAP12 Chain B, with DAP12 Chain B represented in mauve and TREM2 in blue.

We present representative snapshots and contact maps for TREM2 and both chains of the DAP12 dimer from each model (Figure 3). The kinked region around K26 in TREM2 and the DXXXT motif (where D and T represent D16 and T20, respectively, and X represents any amino acid) in DAP12 are known to have a salt bridge interaction. We observed that the middle region (the α-helix of the trimer, spanning residues 14–41 of TREM2 and 6–32 of DAP12, depicted by the light blue rectangle) of the unkinked models (6z0i_TD & AF_TD) of the TREM2 protein makes similar amounts of contact with the two DAP12 chains, while in the kinked model (6z0g_TD), the middle region part of TREM2 only makes significant contact with one chain (Chain A). Therefore, despite the different models having identical amino acid sequences in Chain A and Chain B of DAP12, the kinked and unkinked models of TREM2 exhibit distinct binding preferences.

### Identification of Potential Binding Residues in Three TREM2/DAP12 Complexes

**Figure 4:**
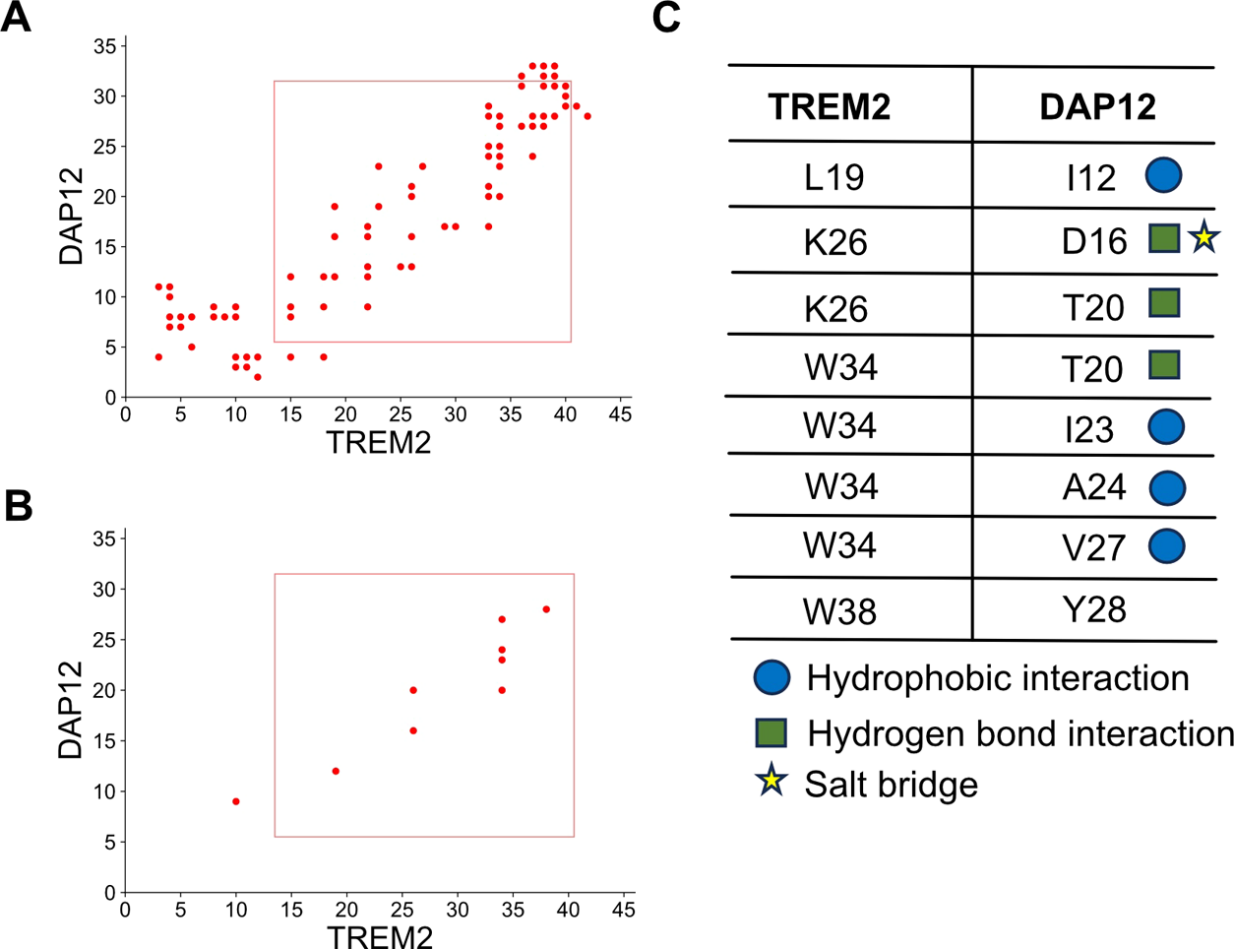
Representation of key binding residues between TREM2 and DAP12. (A) Display of merged total contacts. The rectangle indicates the α-helix region of the TD complex. Part (B) presents the optimized contact map. This optimized contact map uses equal or greater than three times in contact among the total six contact maps. Part (C) illustrates the key binding residue pairs of TREM2 and DAP12. The blue circle represents hydrophobic interactions, the green rectangle signifies hydrogen-bond interactions, and the yellow star denotes salt bridge interactions.

To gain deeper insights into the various binding mechanisms, we constructed contact maps using a 3 Å cutoff distance, involving all atoms including side chain atoms (Figure S6). Contacts persisting for >50% of the total simulation time were considered in the normalized contact map (Figure S7). Summarizing the six contact maps, we illustrated all contacts in Figure 4A. From this data, the most common contacts between residues, which are those that were present in equal or more than half of the six contact maps, were plotted in Figure 4B.

Upon analyzing these pivotal pairs of residues that are in contact in Figure 4B, we inferred that hydrophobic interactions, hydrogen bonds, and salt bridge interactions play crucial roles in the binding of TREM2 to DAP12 (Figure 4C). Furthermore, we produced atomistic contact maps for each of the eight significant residue pairs (Figures S8–S15). These atomistic contact maps help delineate the specific atoms that are in contact with the identified residues.

### Key Interactions in the Binding of TREM2/DAP12

**Figure 5:**
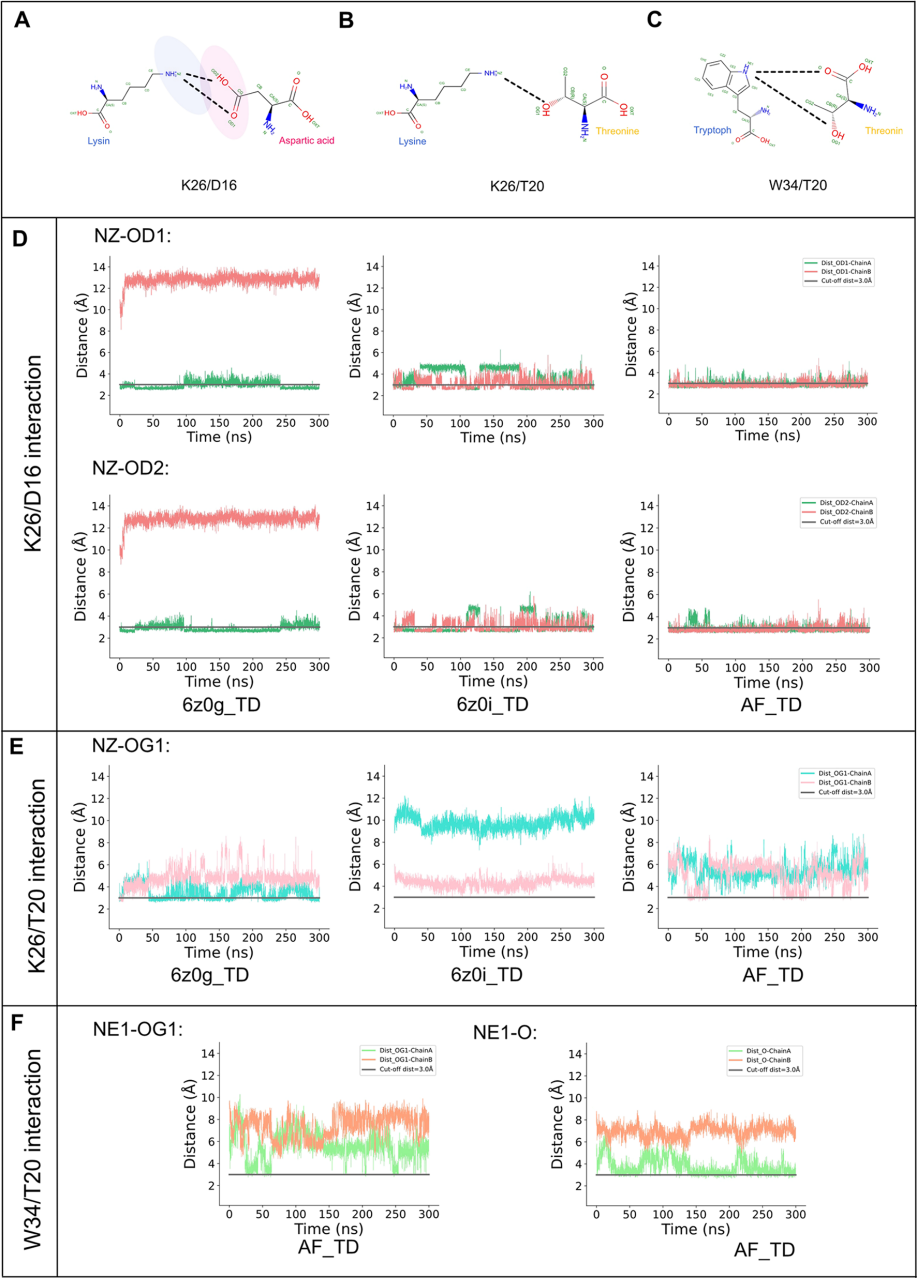
Key hydrogen-bond interactions between TREM2 and DAP12. Binding interaction of K26 of TREM2 with D16 of DAP12. The dotted black lines represent hydrogen bonds between the relevant atoms. The blue oval highlights the positively charged region, while the pink oval highlights the negatively charged region. (B) Interaction of K26 with T20, showing hydrogen bonds between them. (C) Interaction of W34 with T20, depicting the hydrogen bonds between these residues. (D) Hydrogen-bond distances between K26 of TREM2 and D16 of DAP12 in Chain A. The green line represents the OD1 distance, and the red line represents the OD2 distance. The gray line indicates a cutoff distance of 3. TD_OD1 distance is measured between NZ (K26) and OD1 (D16), and TD_OD2 distance is measured between NZ (K26) and OD2 (D16). (E) Hydrogen-bond distances between K26 and T20 in the three systems, measured using NZ (K26) and OG1 (T20). (F) Hydrogen-bond distances between W34 and T20 in the AF_TD system. AF_TD_OG1 represents the distance between NE1 (W34) and OG1 (T20), while AF_TD_O represents the distance between NE1 (W34) and O (T20).

We then identified three different potential hydrogen-bonding partners within the TREM2/DAP12 complex: (i) K26/D16, (ii) K26/T20, and (iii) W34/T20 (Figure 5A–C, respectively). The distances between each of the donors and acceptors within these three pairs are listed in Figure 5.

In the three different models, we observe hydrogen bonds formed that stabilize the salt bridge formed between the K26 residue in TREM2 and the D16 residues in DAP12. In 6z0g_TD, the K26 residue in TREM2 forms a hydrogen bond with the D16 in Chain A while no such hydrogen bond is observed with D16 in Chain B (Figure 5D). Whereas in 6z0i_TD (Figure 5E) and AF_TD (Figure 5F), we observe that there are hydrogen bonds formed by the K26 residue with both D16 residues, and the probability of a hydrogen bond forming to one chain compared to another is approximately the same (Table 1). In all three systems, it is worth noting that while there is a consistent hydrogen bond between the K26 residue in TREM2 and a D16 residue in DAP12, it is found that the actual hydrogen bond alternates between the NZ of K26 and OD1 of D16 and OD2 of D16. Given that 6z0i_TD and AF_TD systems featured less kinked TREM2 structures, it is inferred that the kinked structure of TREM2 (6z0g_TD) results in it only being able to form hydrogen bonds to one chain of DAP12 as opposed to both chains when it is unkinked.

**Table 1:** Hydrogen-Bond Probabilities of TREM2/DAP12 in the Three Systems

system	residue	H-bond	chain A^a^	chain B^b^
6z0g_TD	K26/D16	NZ-OD1	34.74	0
		NZ-OD2	44.57	0
	K26/T20	NZ-OG1	15.10	0.52
	W34/T20	NE1-OG1	0	0
		NE1-O	0	0
6z0i_TD	K26/D16	NZ-OD1	24.85	34.91
		NZ-OD2	60.35	43.03
	K26/T20	NZ-OG1	0	0
	W34/T20	NE1-OG1	0	0
		NE1-O	0	0
AF_TD	K26/D16	NZ-OD1	32.86	33.29
		NZ-OD2	38.49	34.78
	K26/T20	NZ-OG1	0	1.02
	W34/T20	NE1-OG1	0.55	0
		NE1-O	9.08	0

a The hydrogen bonds are calculated between TREM2 and DAP 12 chain A.

b The hydrogen bonds are calculated between TREM2 and DAP 12 chain B.

Unlike hydrogen bonds between the K26 residue of TREM2 and the D16 residues of DAP12, the K26/T20 hydrogen bond is observed primarily in the kinked conformation (6z0g_TD). In 6z0g_TD, we observe that the hydrogen bond is primarily found with one chain of DAP12 (Figure 5E and Table 1). While we do observe the formation of a K26/T20 hydrogen bond in the AF_TD system, it is very transient and, therefore, is not found to have any significant degree.

Finally, we investigated the potential W34-T20 hydrogen-bond pairing. In this case, we found it only in the AF_TD system (Figure 5F and Table 1). Again, we find that, in this case, the hydrogen bond is primarily found between TREM2 and one chain of DAP12.

**Figure 6:**
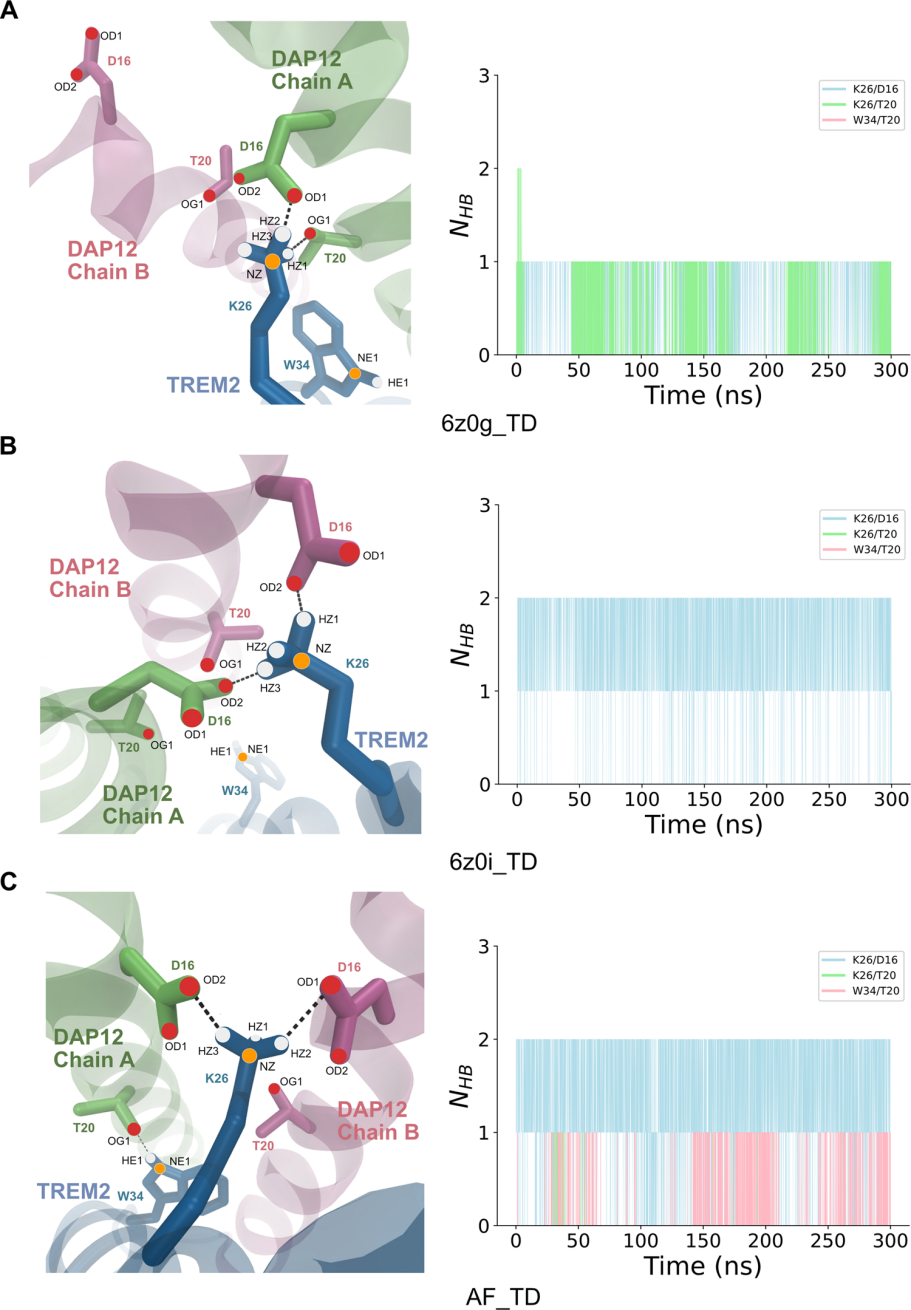
Binding interface of hydrogen-bond interactions in three systems. (A) Left picture displays the hydrogen binding interface of 6z0g_TD. Black-dashed lines represent the observed hydrogen bonds within the time frame. The right picture showcases the count of hydrogen bonds of K26/D16, K26/T20, and W34/T20, colored in blue, green, and pink, respectively. Part (B) illustrates the hydrogen-bond interface and the count of hydrogen bonds in 6z0i_TD. Part (C) demonstrates the hydrogen-bond interface and the count of hydrogen bonds in AF_TD. The gray-dashed line indicates the hydrogen bond of W34/T20.

We selected a representative snapshot (at *t* = 150 ns) to display the binding interface of TREM2 and DAP12 in the three different systems (Figure 6). In the system with the kinked TREM2 (6z0g_TD), the K26 residue of TREM2 binds to the D16 and T20 residues of Chain A of DAP12, forming NZ-HZ2-OD1 and NZ-HZ1-OG1 hydrogen bonds, respectively (Figure 6A). The number of hydrogen bonds (*N*_HB_) is also illustrated in Figure 6A to facilitate a better understanding of the distribution of the various types of hydrogen bonds. Within the 6z0i_TD system, hydrogen bonds are exclusively formed between the K26 and D16 (Figure 6A). This system forms two hydrogen bonds (NZ-HZ1-OD2 & NZ-HZ3-OD2) during the course of the simulation. In the AF_TD system, all three types of hydrogen bonds are detected (Figure 6C). Within the interface, NZ-HZ3-OD2 and NZ-HZ2-OD1 form two hydrogen bonds. Additionally, we display the third hydrogen-bond NE1-HE1-OG1 in W34/T20 in the interface (illustrated as a gray-dashed line) (Figure 6C).

The combined total of hydrogen bonds found in any of the three different models for the TREM2/DAP12 complex is found to be generally two, and in the AF_TD model, occasionally, there is a third hydrogen bond formed (Figure S16A–C).

## Discussion

We understand that TREM2 and DAP12 play crucial roles in Alzheimer’s disease, but the structure of their complex remains elusive to date. Wet laboratory experiments have highlighted the significance of K26 in TREM2, but specific details have remained mysterious. An interesting comparison in exploring the structure of the TREM2/DAP12 complex was observed in the structure of NKG2C (Natural Killer Cell Group 2)/DAP12,^19^ which shares some commonalities with TREM2/DAP12.

However, due to limitations in protein purification, the authors introduced covalent bonds between NKG2C and DAP12, potentially impacting the interaction. In the NKG2C/DAP12 complex, NKG2C has the opposite orientation with DAP12 from the N-terminal to C-terminal, and NKG2C binds DAP12 via K52 of NKG2C and DXXXT(16–20) motif of DAP12.^19^ While in the TREM2/DAP12 complex, TREM2 and DAP12 have the same orientation from the N-terminal to C-terminal, and TREM2 binds DAP12 via D16 of TREM2 and the DXXXT(16–20) motif of DAP12.

Our model precisely utilizes the same sequences in three models and unveils the binding modes of both kinked and unkinked TREM2 structures. Additionally, we accurately determine the hydrogen bonds between TREM2/DAP12 and visualize the changing hydrogen-bond patterns with different models. These findings align with the previous wet laboratory discoveries, elucidating that the significance of the kink is essential for the complex’s functionality. Remarkably, the predicted structure of AlphaFold2 closely resembles the 6z0i_TD structure. There are at least three TREM2 isoforms that have been identified in human brain tissue, with isoform 1, which encodes the full-length protein, being the most abundantly expressed. The mutation associated with Alzheimer’s disease (AD), W34X, in isoform 1 of TREM2 was found earlier,^24^ and interestingly, this mutation is one of the key interaction amino acid pairs we found, making it a notably important discovery.

**Figure 7:**
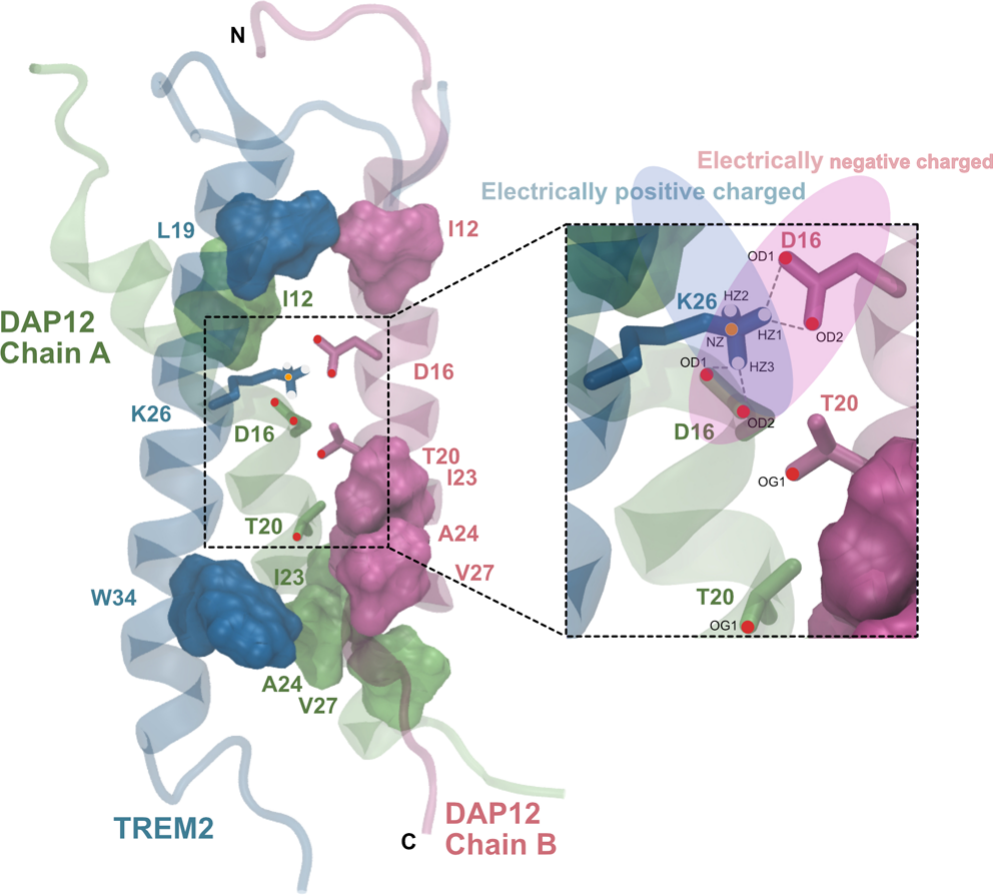
Proposed binding mechanism of TREM2/DAP12. TREM2, DAP12 Chain A, and Chain B are colored in blue, lime, and mauve, respectively. The blue shadow oval region indicates the positively charged area attributed to K26 in TREM2, while red indicates the negatively charged area attributed to D16 in DAP12, depicting the K26/D16 salt bridge interaction. Gray-dashed lines indicate potential hydrogen bonds. Hydrophobbic residue pairs L19/I12, W34/*I*23, W34/A24, and W34/A27 are shown in surface.

Moreover, we have demonstrated the presence of hydrophobic interactions between TREM2 and DAP12 (Figure 4C). Therefore, we generated contact maps for the hydrophobic interactions between L19/I12, W34/*I*23, W34/A24, and W34/A27 (Figures S17–S20, respectively). The AF_TD system exhibited the most prevalent hydrophobic interactions among the three systems, 6z0i_TD showed the least hydrophobic interactions, and 6z0g_TD demonstrated a moderate level of hydrophobic interactions (Figure S21). Considering the interplay of hydrogen bonds, hydrophobic interactions, and salt bridges, we developed an atomic-level TREM2/DAP12 binding model (Figure 7). The snapshot is captured at the 150 ns time frame in the 6z0i_TD system, revealing K26/D16 as the primary contact residue pair capable of forming four hydrogen bonds with NZ in the K26 residue of TREM2 and OD1 and OD2 in the D16 residue in both Chain A and Chain B of DAP12.

It is notable that in the 6z0i_TD system, the hydrogen-bond interaction only existed in K26/D16, and the probability of finding the hydrogen bond is relatively large (85.2 and 77.9% for Chain A and Chain B, respectively, Table 1). A lower probability of the K26/D16 hydrogen bond existing was found in AF_TD (71.4 and 68.1% for Chain A and Chain B, respectively). Also, the AF_TD system has a hydrogen bond between the W34 residue in TREM2 and the T20 residues in DAP12 9.1% of the time and a hydrogen bond between the K26 residue of TREM2 and the T20 residues of DAP12 1.0% of the time. These three types of hydrogen bonds, along with the strong hydrophobic interactions detected in AF_TD (Figure S21), contribute to the stability of the AF_TD system. In contrast, 6z0i_TD exhibits very limited hydrophobic interaction, and only the K26-D16 hydrogen-bond interaction has been observed.

We generated representative snapshots illustrating the orientation of hydrophobic residues in the three systems (Figure S22). In general, the hydrophobic residues in AF_TD predominantly face inward, while those in 6z0i_TD exhibit on the outward-facing side of the proteins, which allows them to interact with the lipids surrounding them. In our proposed model of the TREM2/DAP12 complex, receptor TREM2 primarily binds to the DAP12 dimer through hydrogen bonds formed by K26 in TREM2 and D16 in both chains of DAP12 (Figure 7). Additionally, hydrophobic interactions also play significant roles, with K26/T20 and W34/T20 forming two hydrogen bonds that contribute to system stabilization, albeit to a lesser extent than the K26/D16 interaction.

In addition to providing this picture of the stable TREM2/DAP12 complex, our simulations also provide insight into the mechanisms which play an important role in stabilizing the unbound (kinked) and bound (unkinked) states of TREM2. The kinked state (6z0g_TD) interacts with one chain of DAP12 via a hydrogen bond between its K26 residue and either the D16 residue or the T20 residue on that chain of DAP12. During the binding process, TREM2 undergoes a conformational change such that it is no longer kinked (6z0i_TD & AF_TD), and this allows it to interact with both chains of DAP12. In this bound state, TREM2 then forms stable hydrogen bonds between its K26 residue and both of the D16 residues on either chain of DAP12. We also find evidence of hydrophobic interactions that form between the TREM2 and the two chains of DAP12.

## Methods

### Approach to Build Systems

The 6z0g_TD complex was created by placing the two PDB structures (6z0g^25^ and 2l34(26)) such that there were 6 Å between the K26 residue of TREM2 and the two D16 residues of DAP12, respectively. This was done using the relative orientation of the TREM2 and DAP12 that were determined using HADDOCK.^27,28^ The 6z0i_TD complex was set up using the same method, and AF_TD was built with ColabFold^29,30^ v1.5.2 using the same sequence as 6z0g_TD and 6z0i_TD (the sequence is shown in Table S4). Each system was then equilibrated by using all-atom molecular dynamics simulations for 100 ns. The systems utilized in this manuscript were generated through the use of the CHARMM-GUI Membrane Builder^31^ and the MARTINI Maker^32^ for all-atom (AA) and coarse-grained (CG) simulation, respectively. The membrane consists of POPC and cholesterol in a ratio of 80:20. The solvent employed in every simulation was water, and to maintain a neutral charge and a 0.15 M salt concentration, Na^+^ and Cl^–^ ions were added to the system.

### Coarse-Grained Molecular Dynamics Simulations

We carried out 200 μs CG simulations to gain stable systems in a POPC/cholesterol lipid bilayer. These CG simulations were carried out using GROMACS^33^ version 2019.2 on the Young cluster. MARTINI22P force field^34^ was employed in this system, and each lipid membrane system was first minimized and then equilibrated at a temperature of 310.15 K. The MARTINI22P model groups three to five heavy (non-hydrogen) atoms into each interacting bead, with different bead types representing various combinations of grouped atoms and chemical environments. There are four groups of bead type: charged, polar, nonpolar, and polar, each with five distinct types distinguished by their polar affinity or tendency to form hydrogen bonds in the MARTINI22P force field. The details of the CG simulations of different TREM2/DAP12 systems are shown in Table 2. We carried out three replicas of each system.

**Table 2:** Details of Each of the CG Simulation Systems

system	*n*_atoms_	*n*_lipids_	*n*_waters_	*n*_ions_	final box size (nm)
6z0g_TD^*a*^	9287	210	5463	125	100.6 × 100.6 × 112.0
6z0i_TD	9336	210	5510	127	100.6 × 100.6 × 113.0
AF_TD	11 320	315	7398	167	101.0 × 101.0 × 134.7

### All-Atom Molecular Dynamics Simulations

After 200 μs CG simulations, we converted the CG simulation to all-atom simulations using CHARMM-GUI Martini to All-atom Converter^31,35^ and ran another 300 ns. We utilized GROMACS 2020.3 for these AA simulations on the cluster. Our AA simulations utilized the same POPC/cholesterol bilayer, with lipids modeled using the CHARMM36 force field^36,37^ and CHARMM-modified TIP3P water model.^38^ To ensure accurate results, each lipid membrane system was first minimized and then equilibrated at a temperature of 310.15 K and a pressure of 1 bar according to the simulation protocol prescribed by CHARMM-GUI.^38,39^ The same simulation procedure was applied to all of the systems. It commenced with an initial energy minimization step using the steepest descent algorithm, followed by a 125 ps simulation within the NVT ensemble, where the Nosè–Hoover thermostat was employed to regulate the temperature (set at 310.15 K) with a time step of 1 fs (fs). Subsequently, 300 ns production simulations were executed within the NPT ensemble, utilizing both the Nosè–Hoover thermostat and the Parrinello–Rahman barostat to maintain a constant temperature of 310.15 K and a pressure of 1 atm (atm). Throughout these simulations, hydrogen-containing bonds were kept fixed using the LINCS algorithm.^40^ Periodic boundary conditions were employed across all dimensions throughout all simulations, with a time step of 1 fs utilized for both the equilibration and production simulations. The cutoff used for the calculation of Coulombic and Lennard-Jones (LJ) forces was 12 Å, and a switching function reduced the LJ interactions to zero from an inner cutoff of 10 Å. Table 3 displays the AA simulation details of the TREM2/DAP12 complex. Three replicas were performed.

**Table 3:** Details of Each of the AA Simulation Systems

system	*n*_atoms_	*n*_lipids_	*n*_waters_	*n*_ions_	final box size (nm)
6z0g_TD	77 286	220	16214	87	85.7 × 85.7 × 112.3
6z0i_TD	78 446	220	16600	89	85.6 × 85.6 × 115.2
AF_TD	94 204	225	21640	117	86.0 × 86.0 × 136.0

### Molecular Dynamics Simulation Analysis

The analysis of all data was performed using in-house Python (3.10) scripts, which make wide use of the MDAnalysis package^41,42^ and are visualized using Matplotlib.^43^ Furthermore, all simulations conducted in this study were performed under periodic boundary conditions, with the resulting trajectories viewed through the utilization of VMD^44^ 1.9.3. The kinked angles were calculated using the C_α_ atoms in TREM2, specifically utilizing residues A20 and L24 on TREM2 as the first vector and residues A32 and A36 as the second vector. The contact map presented in Figure 3 was generated using the C_α_ atoms between TREM2 and DAP12 with a cutoff distance set at 11 Å. The hydrogen-bond possibility was computed utilizing the Hydrogen Bond Analysis^45^ function within MDAnalysis and was executed using an in-house Python script. The cutoff distance for the hydrogen bond is set to 3 Å, and the cutoff angle is set to 150°.

## Supporting Information Available

Supporting Information includes (i) thorough description of all analysis conducted for our simulations, (ii) plots of the contacts as a function of time and residue-resolution contact maps of the TREM2/DAP12 complexes studied, (iii) information regarding the AlphaFold predicted structure used in this study, (iv) atomistic-resolution contact maps of the key interactions in the complexes and, and (v) amino acid sequences of the TREM2 and the two chains of DAP12 (PDF)
