# Supplementary material for: Unraveling the Molecular Dance: Insights into TREM2/DAP12 Complex Formation in Alzheimer’s Disease through Molecular Dynamics Simulations
Source: ACS Omega. 2024 Jun 21;9(26):28715–25. doi: 10.1021/acsomega.4c03060 (PMC11223195; doi:10.1021/acsomega.4c03060)
Supplement: Supplementary file 1 — ao4c03060_si_001.pdf [file ao4c03060_si_001.pdf]

Supporting Information

Unraveling the Molecular Dance: Insights into TREM2/DAP12 Complex Formation in Alzheimer's Disease through Molecular Dynamics Simulations

Zhiwen Zhong,[†,‡] Martin Ulmschneider,[*,‡] and Christian D. Lorenz[*,†]

†*Biological Physics and Soft Matter Group, Department of Physics, King's College London, London, WC2R 2LS, UK*

‡*Department of Chemistry, King's College London, London, SE1 1DB, UK*

E-mail: martin.ulmschneider@kcl.ac.uk; chris.lorenz@kcl.ac.uk

This PDF file includes:

Supporting text

Figure S1 to S23

Tables S1 to S4

Supporting Information Text

Stability check of the three systems. Figure S1 depicts the total potential contacts between TREM2 and DAP12. The data spans four different timescales (25 - 75 ns, 100 - 150 ns, 175 - 225 ns, and 250 - 300 ns) within a 300 ns all-atom (AA) simulation, comprising 50 ns simulation intervals. Potential contacts are assessed based on C_α atom distances between TREM2 and DAP12 that are less than 11.0 Å. In the case of 6z0g_TD, the potential contacts for Chain A occur between 100 - 150 ns, while those for Chain B span 70 - 150 ns. For 6z0i_TD, the potential contacts for Chain A remain relatively stable throughout the simulation, reaching around 100 at the conclusion. In contrast, the potential contacts for Chain B fluctuate significantly between 25 - 75 ns and stabilize toward the end of the simulation, also reaching a count of about 100. In the case of AF_TD, the potential contacts exhibit a slight decrease and eventually reach about 100 in both chains.

We used the potential contact maps to assess the stability of the three systems. In the 6z0g_TD system (Figure S2), the potential contact maps for both Chain A (Figure S2A) and Chain B (Figure S2B) of DAP12 exhibit a high degree of similarity, suggesting a stable state in the complex.

In the 6z0i_TD system, the potential contact maps for Chain A (Figure S3A) of DAP12 are largely similar. However, minor differences are noticed in the 175 - 225 ns period for 6z0o_TD Chain B (Figure S3B). These discrepancies are observed in the N-terminal regions of the TREM2/DAP12 trimer, known for containing unstructured loops. Our focus on the α-helix region indicates acceptable consistency in the middle region contact maps across all three contact maps.

As for the AF_TD system, the potential contact maps exhibit consistency across the 25 - 75 ns, 100 - 150 ns, and 175 - 225 ns periods (Figure S4). However, in the 250 - 300 ns period, a decrease in contacts is noted in both chains within the N-terminal region of TREM2/DAP12, while the middle region interactions remain stable. These observations indicate a stable interaction within the TREM2 and DAP12 transmembrane domain (TMD)

post the coarse-grained simulations.

Confidence of the TREM2/DAP12 complex generated by AlphaFold2. Fig S5 shows the confidence of the AlplaFold2 predicted structures.

Contact maps of TREM2/DAP12 complex. Figure S6 illustrates the contact maps of TREM2/DAP12 using a cutoff distance of 3 Å for all atoms, including side chain atoms. Meanwhile, Figure S7 depicts the regenerated potential contact maps of the TREM2/DAP12 complex. It uses an indexing system where a dot is placed in the contact map if the contact time exceeds 50 % of the total time.

Contact maps of key residues in TD complex. Figure S8 - S15 display the contact maps of key residue pairs, comprising K26/D16, K26/T20, W34/T20, L19/I12, W34/I23, W34/A24, W34/V27, and W38/Y28, respectively.

Detailed hydrogen bond possibilities of the TREM2/DAP12. Table S1 - S3 shows the hydrogen bond possibilities.

Mergerd hydrogen bond interaction. Figure S16 shows the merged hydrogen bond interaction.

Hydrophobic interaction of key residues. Figure S17, S18, S19, and S20 display the residue pairs involved in hydrophobic interactions, namely L19/I12, W23/I23, W34/A24, and W34/V27, respectively. Figure S21 illustrates the distance between these residue pairs to determine hydrophobic interactions. In this analysis, a hydrophobic interaction is defined when the distance between any carbon atoms within a particular residue and another residue is below 5 Å. Figure S22 shows the facing direction of hydrophobic residues in three systems.

The snapshots were captured in 150 ns.

Sequence of three systems. Table S4 shows the amino acid sequence of our built systems.

Stability check of the three systems. Figure S1 depicts the total potential contacts between TREM2 and DAP12. The data spans four different timescales (25 - 75 ns, 100 - 150 ns, 175 - 225 ns, and 250 - 300 ns) within a 300 ns all-atom (AA) simulation, comprising 50 ns simulation intervals. Potential contacts are assessed based on C-α atom distances between TREM2 and DAP12 that are less than 11.0 Å. In the case of 6z0g_TD, the potential contacts for TD_Chain A occur between 100 - 150 ns, while those for TD_Chain B span 70 - 150 ns. For 6z0i_TD, the potential contacts for Chain A remain relatively stable throughout the simulation, reaching around 100 at the conclusion. In contrast, the potential contacts for Chain B fluctuate significantly between 25 - 75 ns and stabilize toward the end of the simulation, also reaching a count of about 100. In the case of AF_TD, the potential contacts exhibit a slight decrease and eventually reach about 100 in both chains.

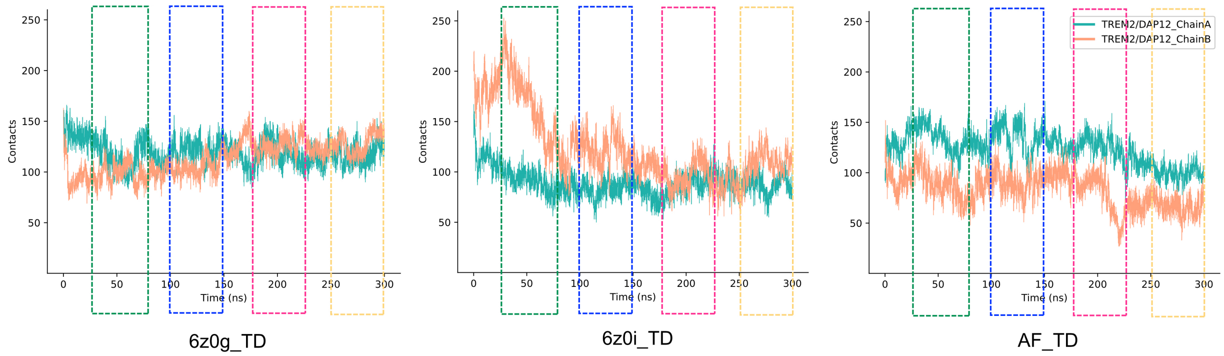

Figure S1: Total contacts across the three systems. Distinctly coloured rectangles indicate various time scales: green, blue, red, and yellow respectively represent 25 - 75 ns, 100 - 150 ns, 175 - 225 ns, and 250 - 300 ns. Contacts are colour-labelled according to the different chains within DAP12; sea-green designates the interaction of TREM2 and DAP12 Chain A, while orange represents the interaction of TREM2 and DAP12 Chain B.

We used the potential contact maps to assess the stability of the three systems. In the 6z0g_TD system (Figure S2), the potential contact maps for both Chain A (Figure S2A) and Chain B (Figure S2B) of DAP12 exhibit a high degree of similarity, suggesting a stable state in the complex.

In the 6z0i_TD system, the potential contact maps for Chain A (Figure S3A) of DAP12 are largely similar. However, minor differences are noticed in the 175-225 ns period for 6z0o_TD_Chain B (Figure S3B). These discrepancies are observed in the N-terminal regions of the TREM2/DAP12 trimer, known for containing unstructured loops. Our focus on the α-helix region indicates acceptable consistency in the middle region contact maps across all three contact maps.

As for the AF_TD system, the potential contact maps exhibit consistency across the 25 - 75 ns, 100 - 150 ns, and 175 - 225 ns periods (Figure S4). However, in the 250 - 300 ns period, a decrease in contacts is noted in both chains within the N-terminal region of TREM2/DAP12, while the middle region interactions remain stable. These observations indicate a stable interaction within the TREM2 and DAP12 transmembrane domain (TMD) post the coarse-grained simulations.

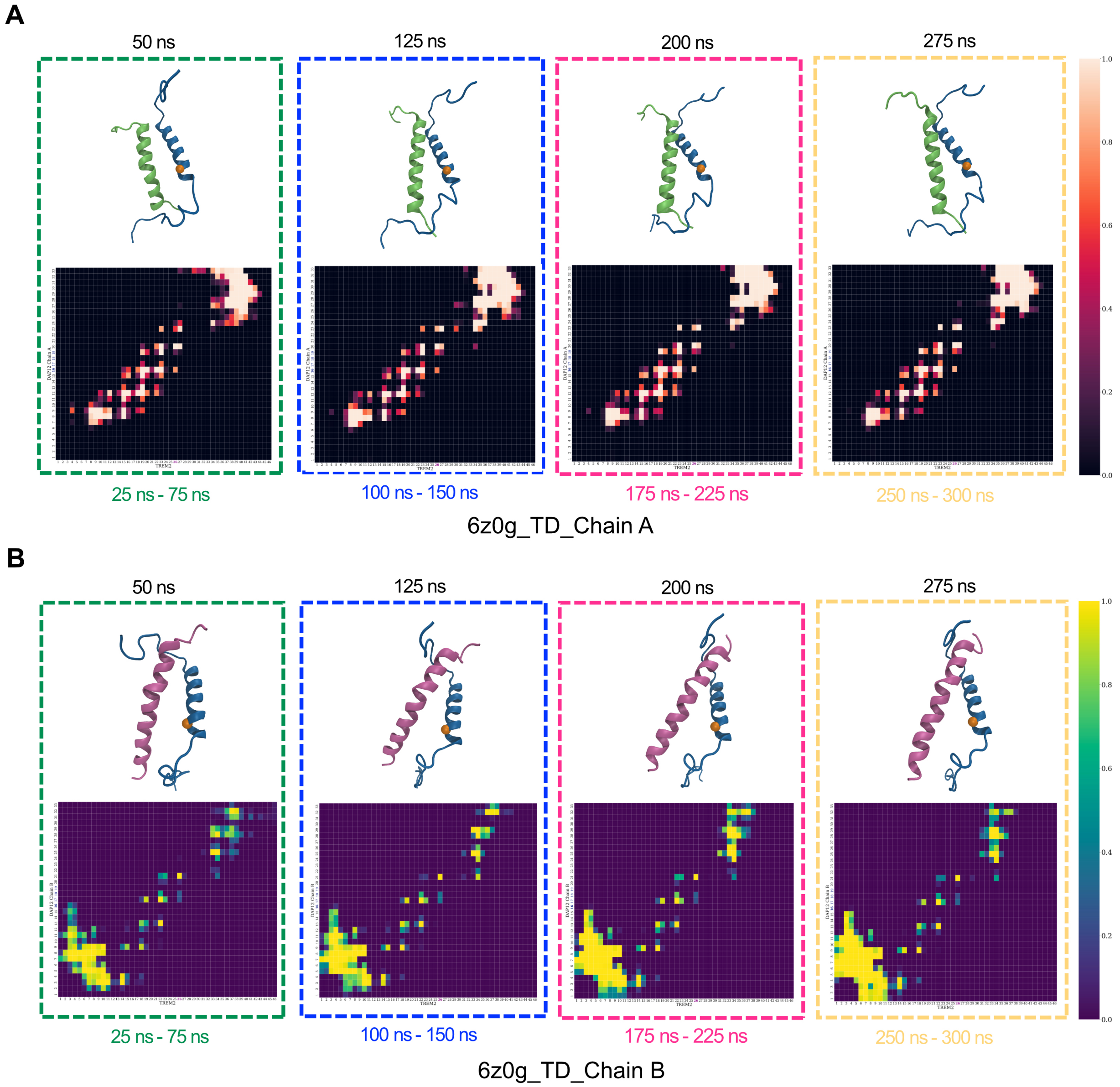

Figure S2: 3D structure snapshots and potential contact maps of 6z0g_TD. (A) The four 3D structures were captured at different times (50 ns, 125 ns, 200 ns, 275 ns) and are represented by differently coloured rectangles, consistent with Figure S1. The blue-coloured α-helix represents TREM2, while the green-coloured α-helix shows DAP12 Chain A. The orange sphere denotes the C-α atom of K26 in TREM2. The potential contact maps depict normalized contact numbers, with black indicating 0 and white indicating 1. These contact maps were calculated over four different timescales (25 - 75 ns, 100 - 150 ns, 175 - 225 ns, and 250 - 300 ns), consistent with Figure S1. (B) In the 3D structure screenshots, the blue-coloured α-helix represents TREM2, while the red-coloured α-helix shows DAP12 Chain B. The contact maps were normalized to 0 and 1, represented by deep blue and yellow, respectively.

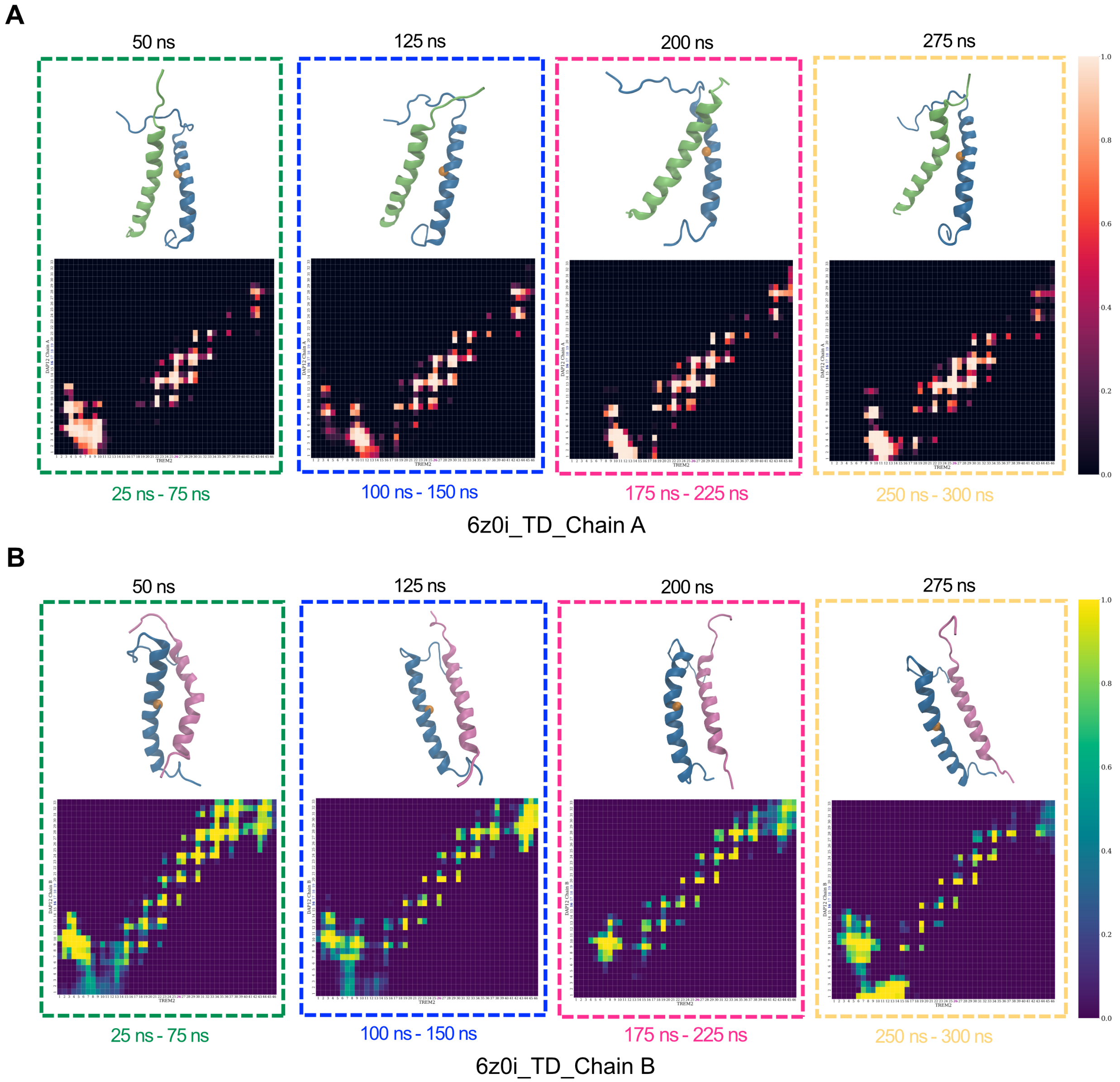

Figure S3: 3D structure screenshots and potential contact maps of 6z0i_TD. (A) and (B) are consistent with Figure S2.

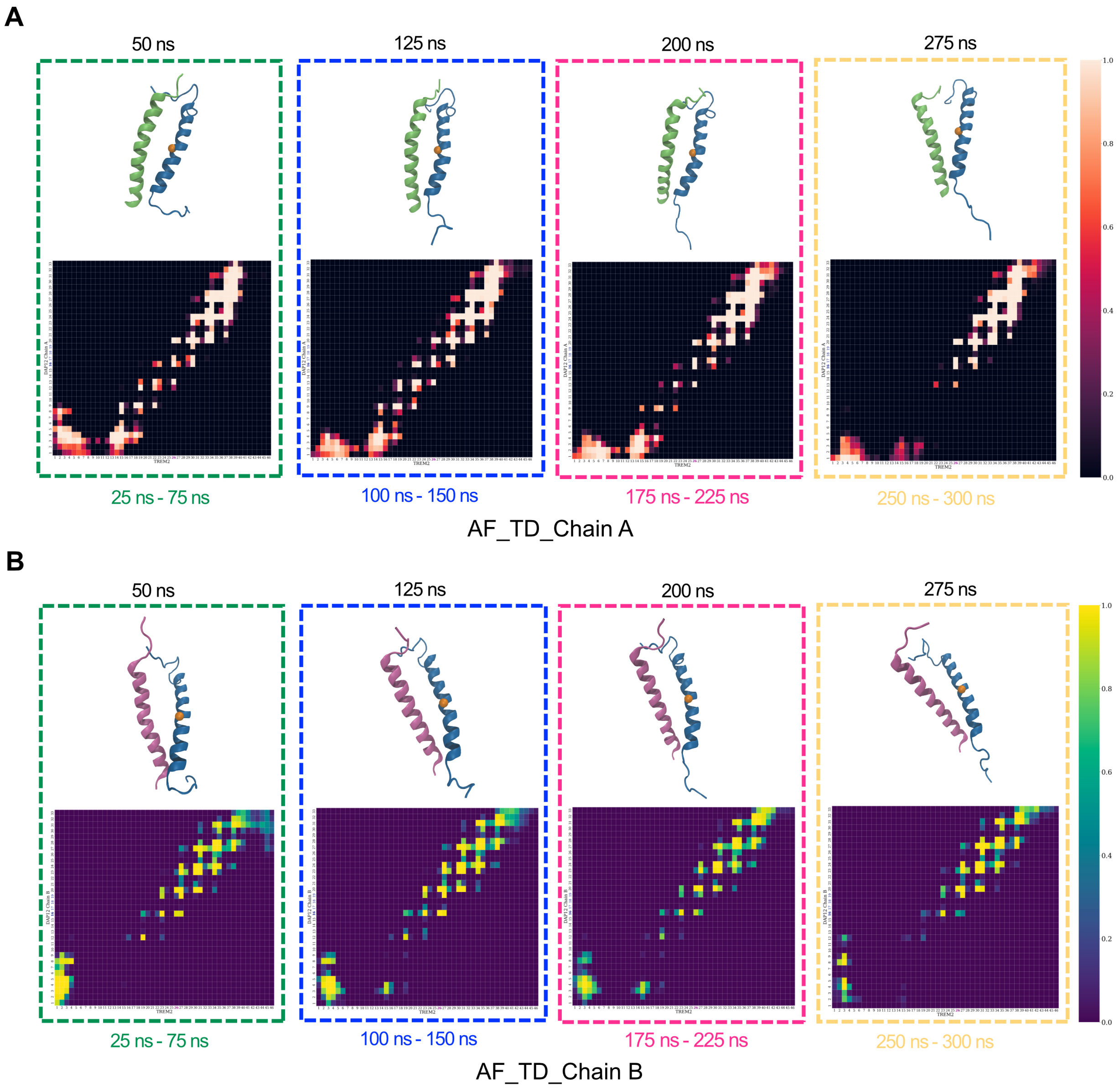

Figure S4: 3D structure screenshots and potential contact maps of AF_TD. (A) and (B) are consistent with Figure S2.

AlphaFold2 predicted structures

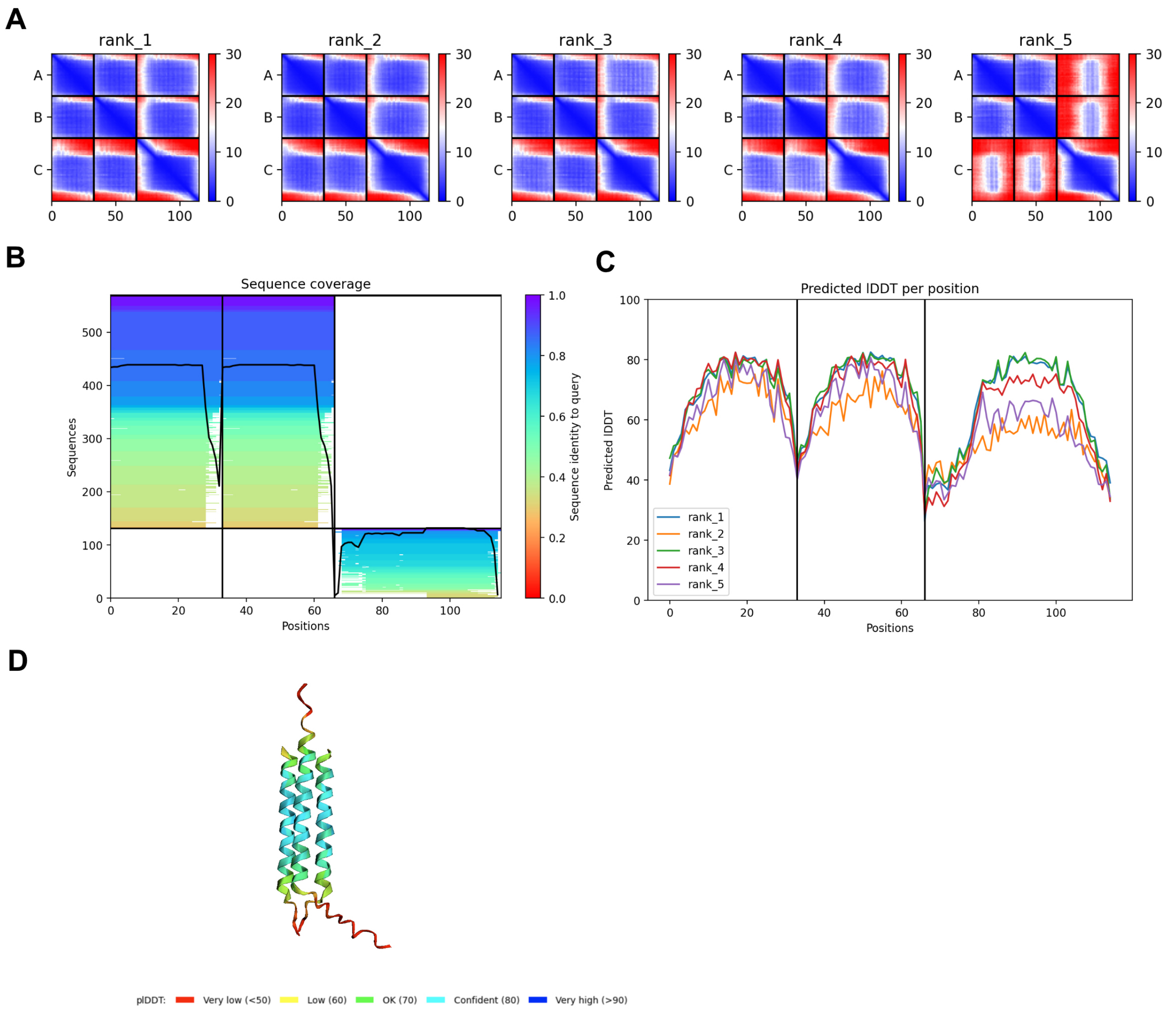

Figure S5: Confidence of AlphaFold2 predicted structures. (A) PAE plots featuring two domains with low conformation confidence. Five different models are run, rank_1 to rank_5 respectively. (B) The multiple sequence alignment is summarised as a heatmap. The heat map representation of the MSA indicates all sequences mapped to the input sequences. The colour scale indicates the identity score, and sequences are ordered from top (largest identity) to bottom (lowest identity). White regions are not covered, which occurs with sub-sequence entries in the database. The black line qualifies the relative coverage of the sequence with respect to the total number of aligned sequences. (C) The predicted IDDT (I-Domain Distance Test)) per residue for the 5 models was obtained after an AlphaFold2 job. (D) The predicted 3D structure of AlphaFold2 is coloured in predicted LDDT (Local Distance Difference Test).

Contact maps of TREM2/DAP12 complex

Figure S6 illustrates the contact maps of TREM2/DAP12 using a cutoff distance of 3Å for all atoms, including side chain atoms. Meanwhile, Figure S7 depicts the regenerated potential contact maps of the TREM2/DAP12 complex. It uses an indexing system where a dot is placed in the contact map if the contact time exceeds 90% of the total time.

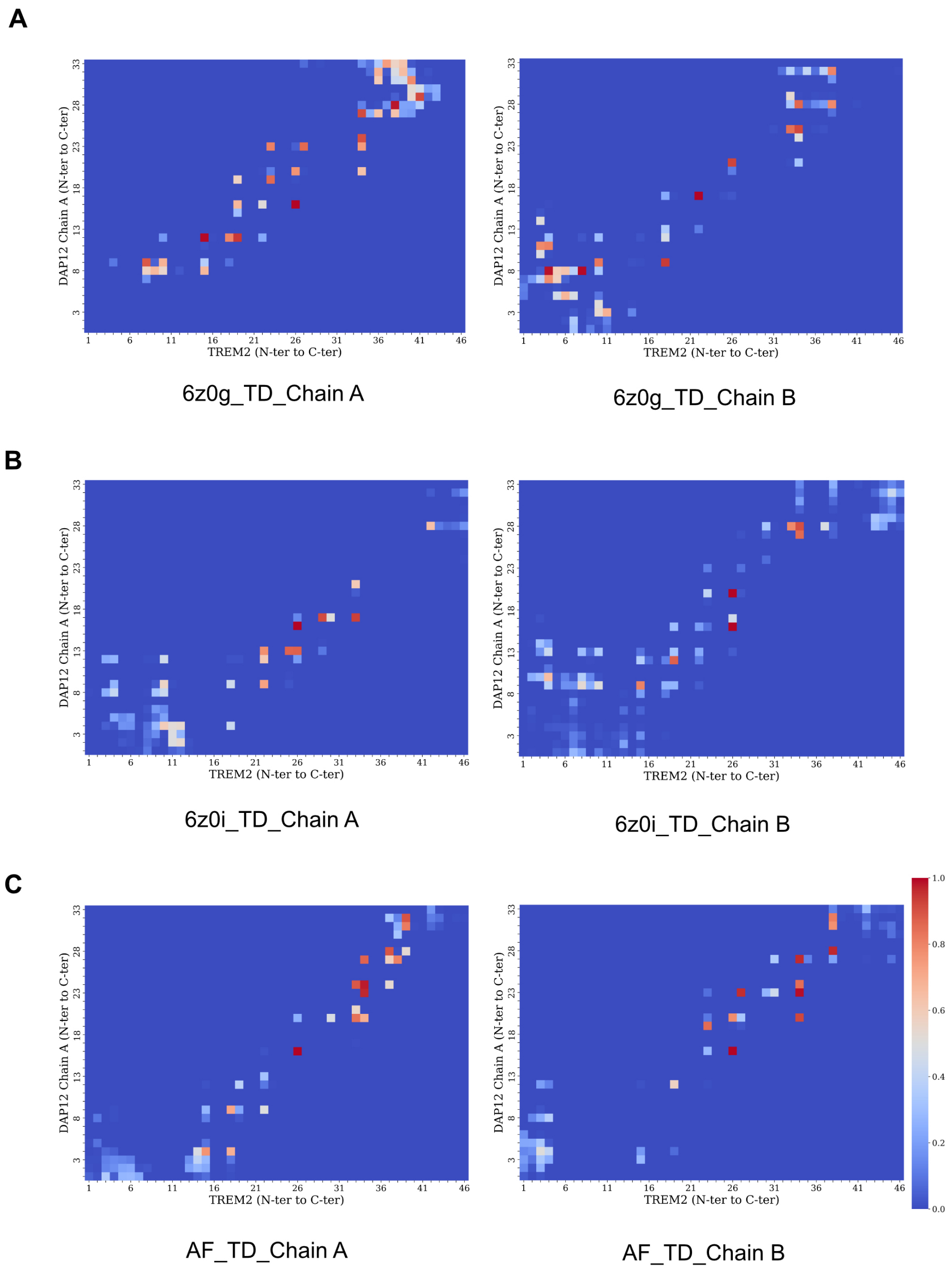

Figure S6: Contact maps of TREM2/DAP12 complex. (A) The contact maps of the 6z0g_TD system between TREM2 and DAP12 Chain A or Chain B (left and right, respectively). (B) The contact maps of 6z0i_TD system between TREM2 and DAP12 Chain A or Chain B (left and right, respectively). (C) The contact maps of the AF_TD system between TREM2 and DAP12 Chain A or Chain B (left and right, respectively).

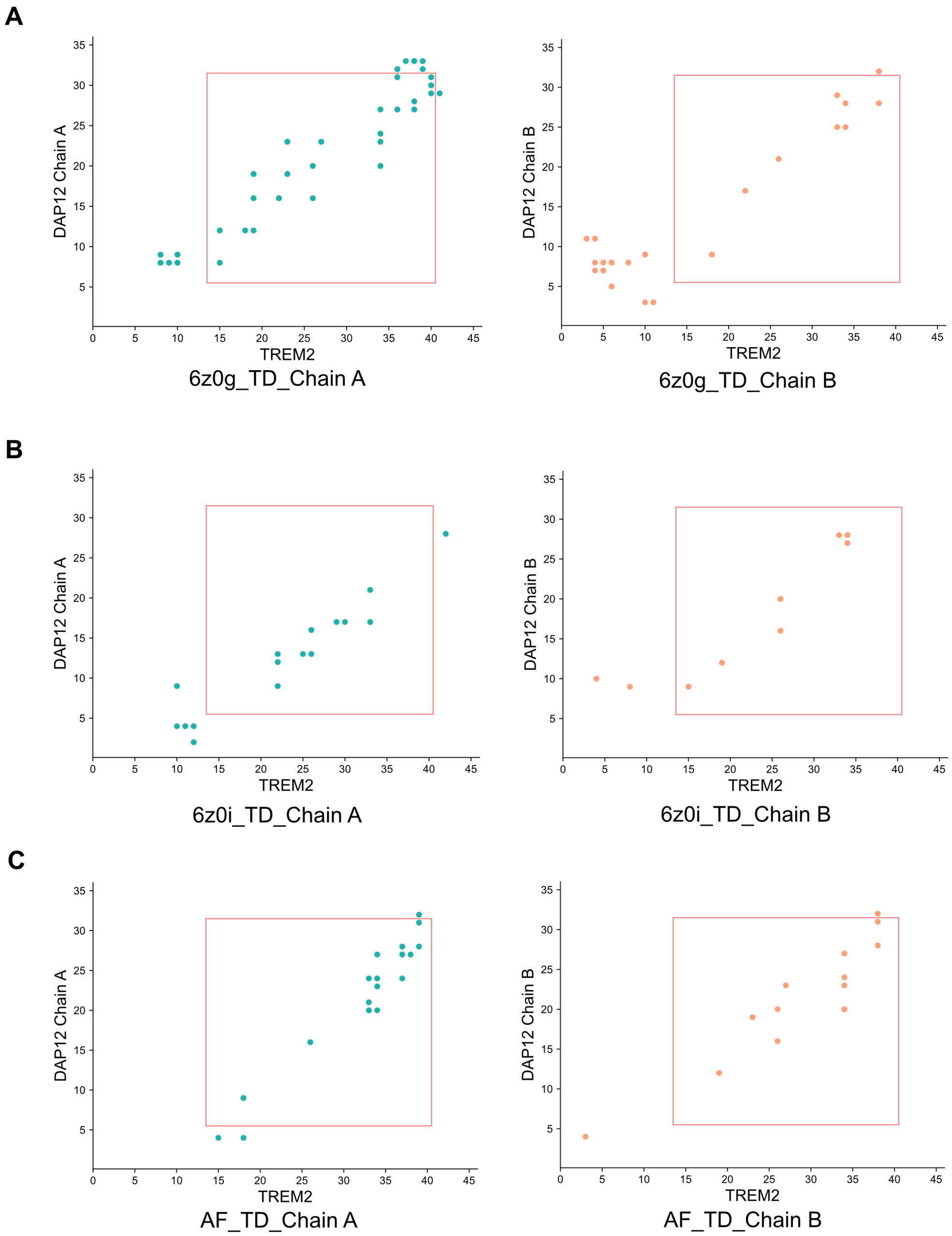

Figure S7: Revised potential contact maps. These calculated potential contact maps were generated using 300 ns trajectory data, and the distance between Cα atoms was below 11 Å for no less than 90% of the whole simulation time. (A) shows the calculated contact maps of 6z0g_TD. The contacts between DAP12_Chain A and TREM2 are in green, and between DAP12_Chain B and TREM2 are in orange. The red rectangle represents the α-helix, and residues in the rectangle indicate the residues in the α-helix, while those outside are in the loop region. (B) shows the calculated contact maps of 6z0i_TD. (C) shows the calculated contact maps of AF_TD.

Contact maps of key residues in TD complex

Figures S8 to S15 display the contact maps of key residue pairs, comprising K26/D16, K26/T20, W34/T20, L19/I12, W34/I23, W34/A24, W34/V27, and W38/Y28, respectively. The range 0-1 in the sidebar shows the normalised possibilities.

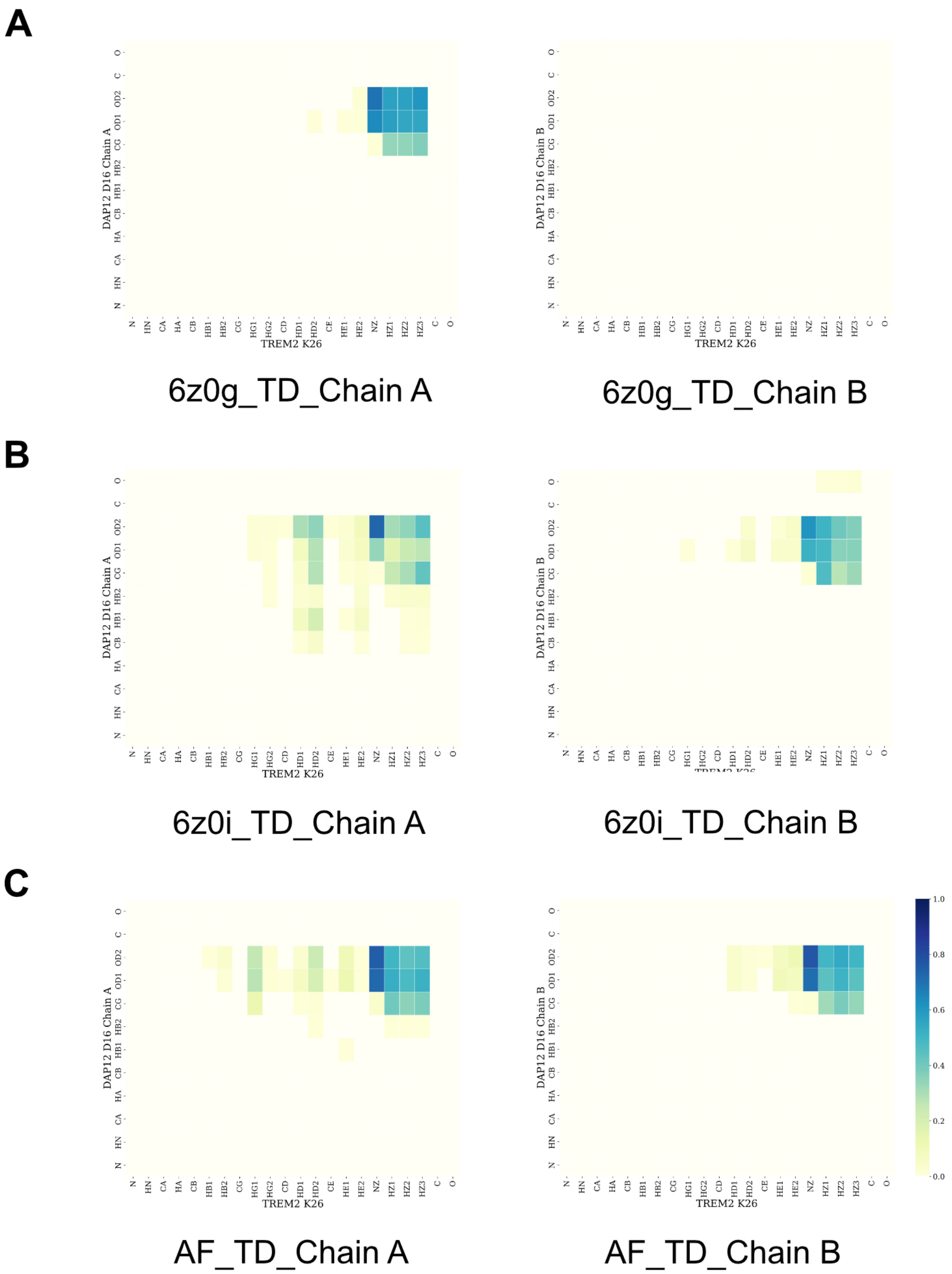

Figure S8: Contact maps of L19/I12 residues. (A) The contact maps of L19/I12 residues in 6z0g_TD system. (B) The contact maps of L19/I12 residues in 6z0i_TD system. (C) The contact maps of L19/I12 residues in AF_TD system.

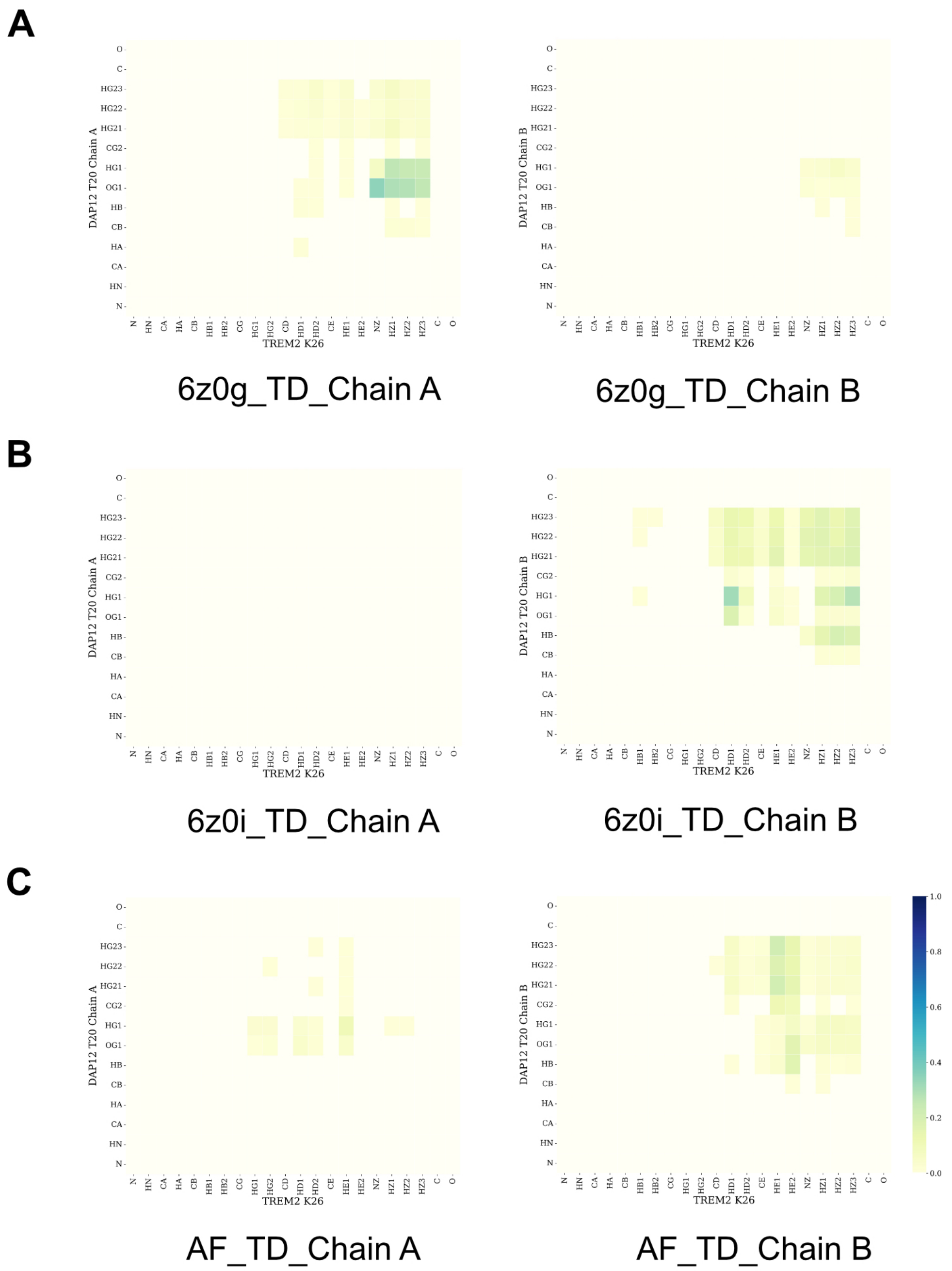

Figure S9: Contact maps of K26/D16 residues. (A) The contact maps of K26/D16 residues in 6z0g_TD system. (B) The contact maps of K26/D16 residues in 6z0i_TD system. (C) The contact maps of K26/D16 residues in AF_TD system.

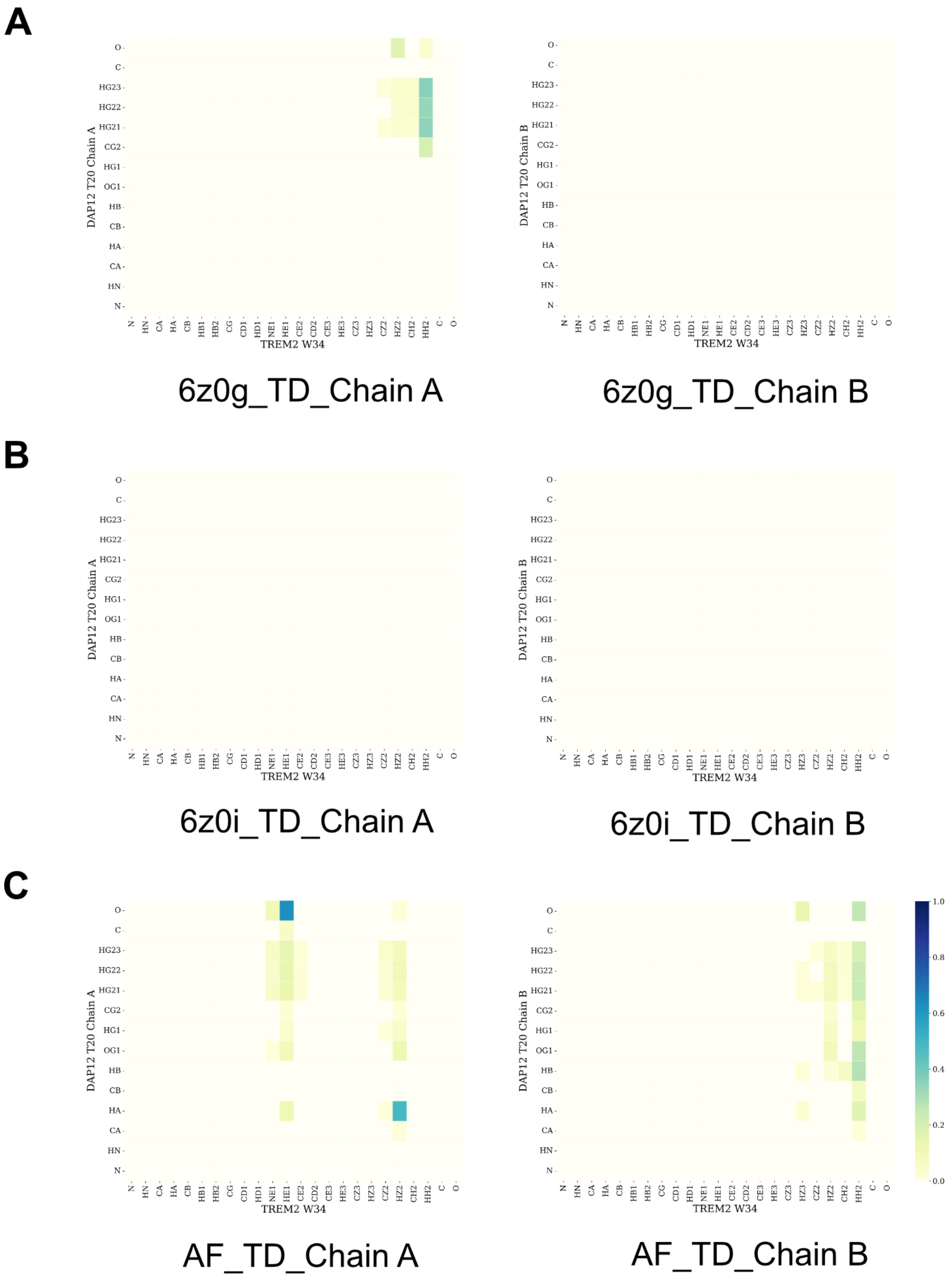

Figure S10: Contact maps of K26/T20 residues. (A) The contact maps of K26/T20 residues in 6z0g_TD system. (B) The contact maps of K26/T20 residues in 6z0i_TD system. (C) The contact maps of K26/T20 residues in AF_TD system.

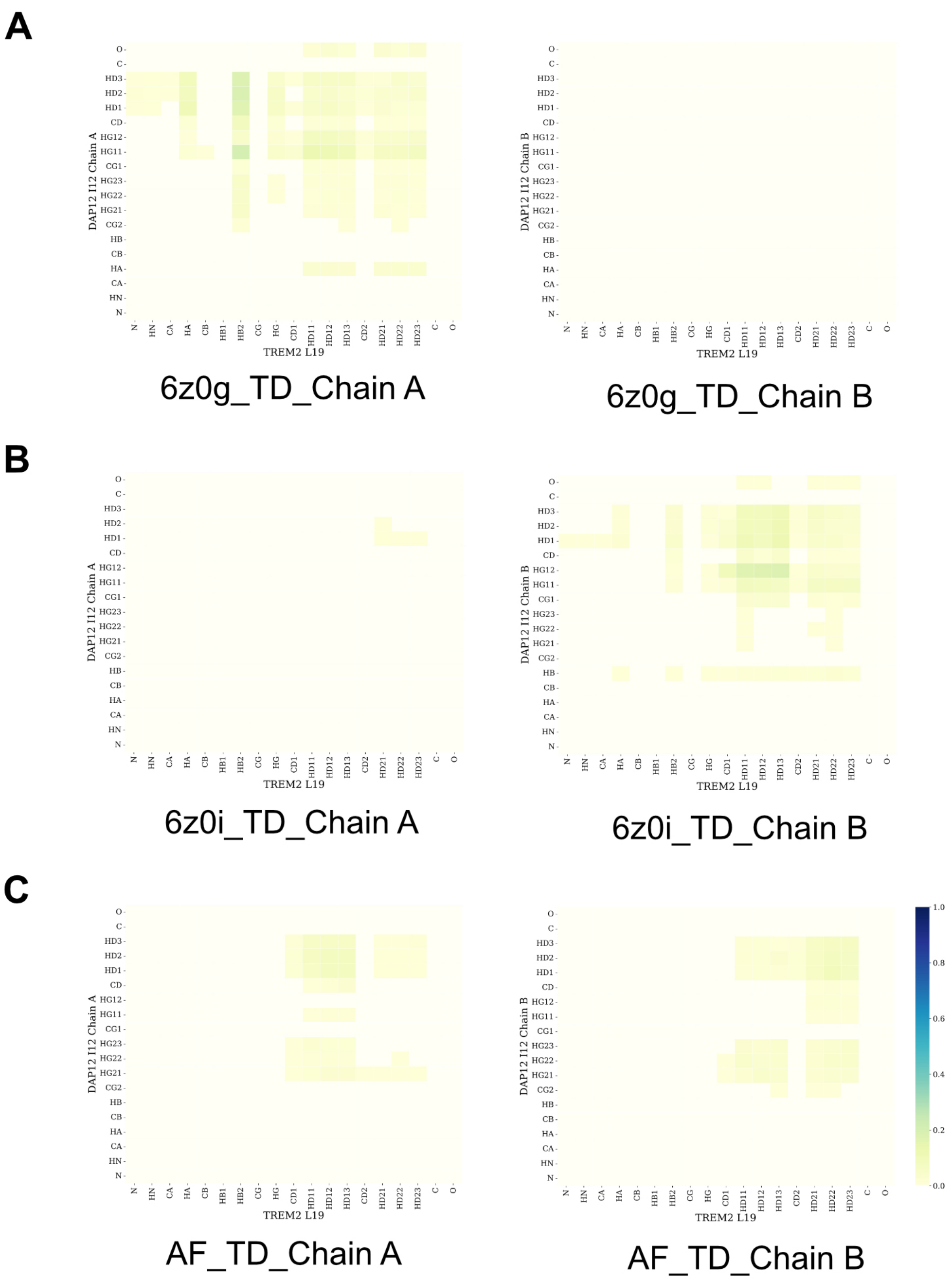

Figure S11: Contact maps of W34/T20 residues. (A) The contact maps of W34/T20 residues in 6z0g_TD system. (B) The contact maps of W34/T20 residues in 6z0i_TD system. (C) The contact maps of W34/T20 residues in AF_TD system.

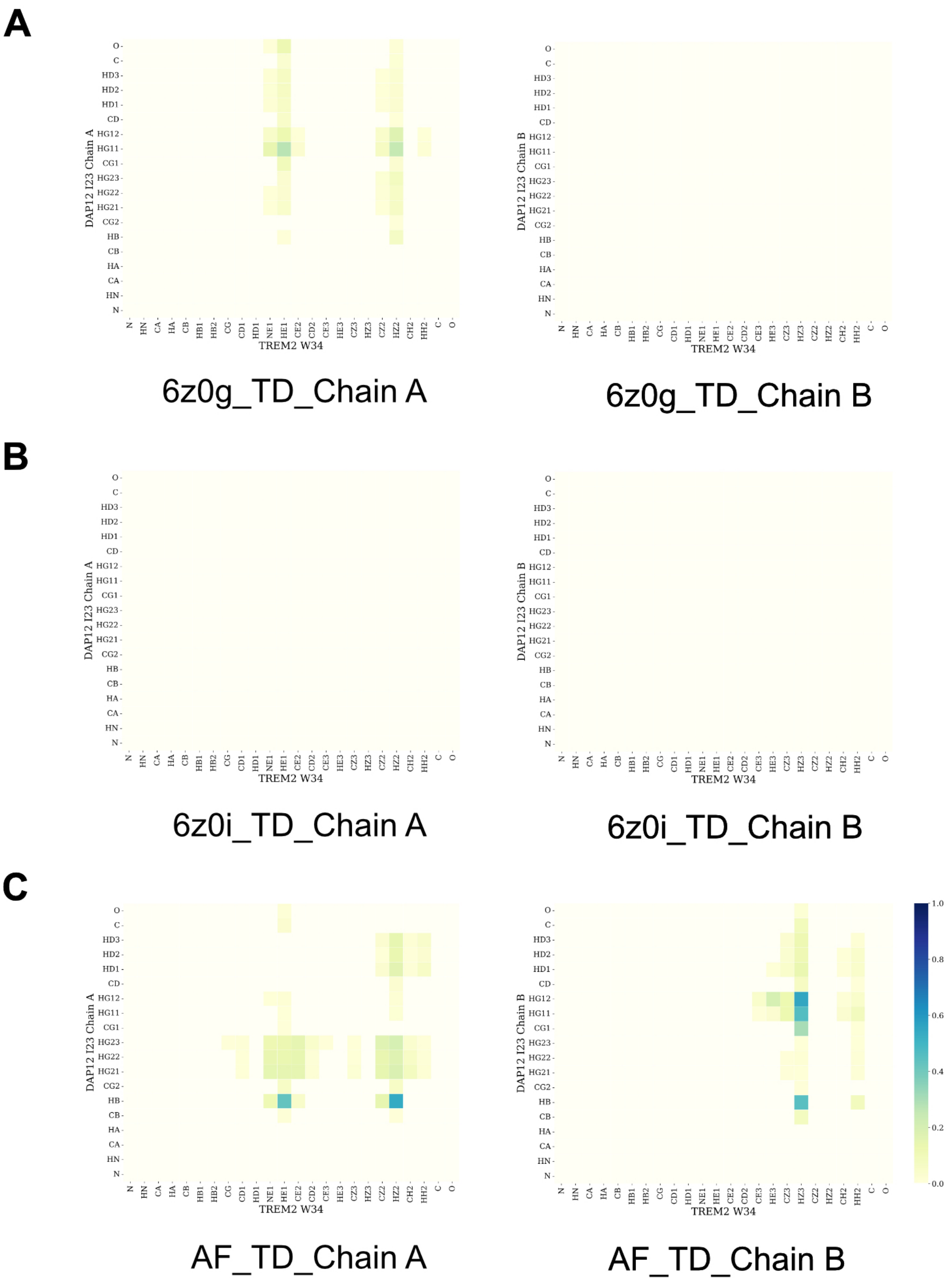

Figure S12: Contact maps of W34/I23 residues. (A) The contact maps of W34/I23 residues in 6z0g_TD system. (B) The contact maps of W34/I23 residues in 6z0i_TD system. (C) The contact maps of W34/I23 residues in AF_TD system.

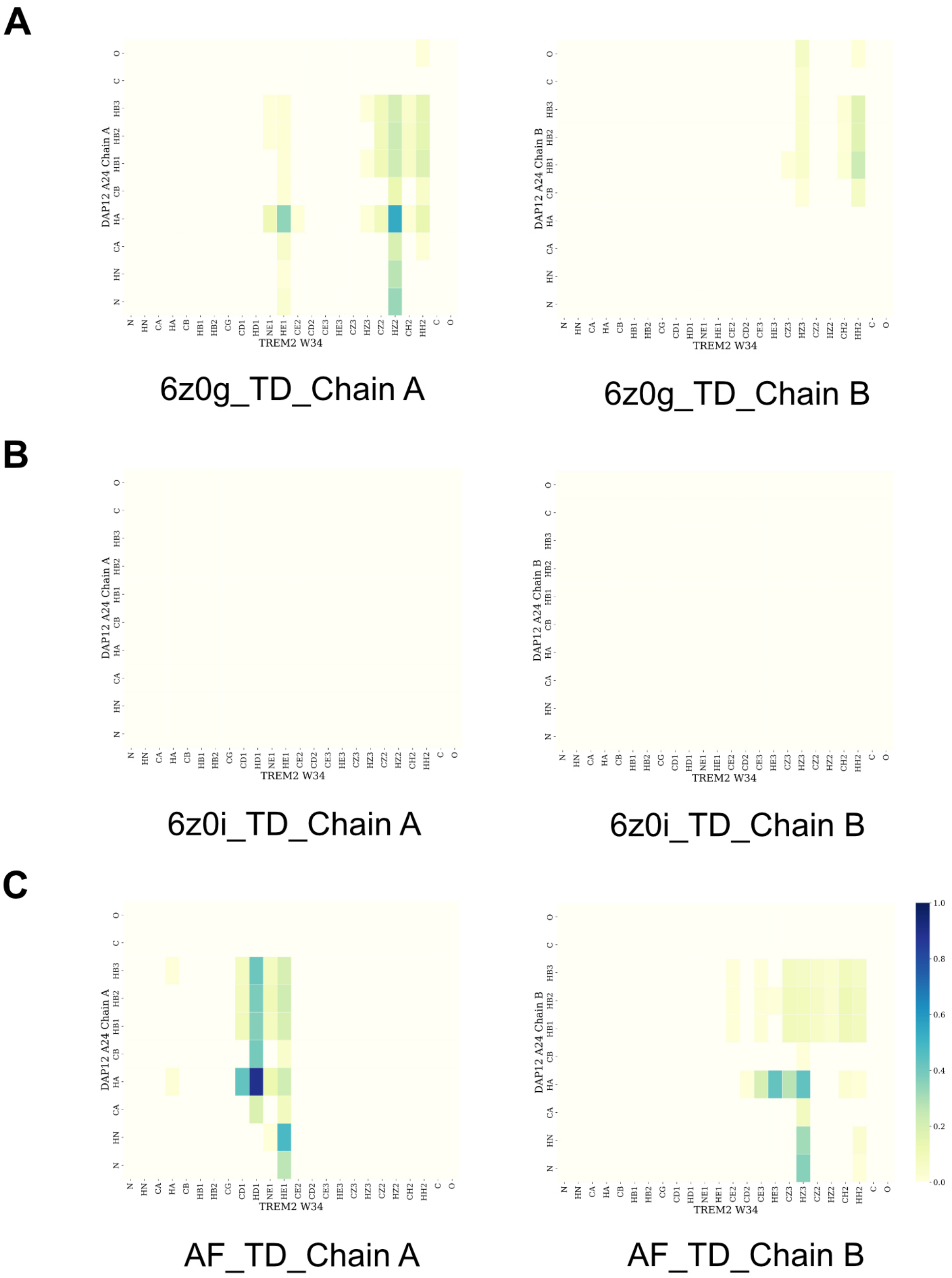

Figure S13: Contact maps of W34/A24 residues. (A) The contact maps of W34/A24 residues in 6z0g_TD system. (B) The contact maps of W34/A24 residues in 6z0i_TD system. (C) The contact maps of W34/A24 residues in AF_TD system.

A

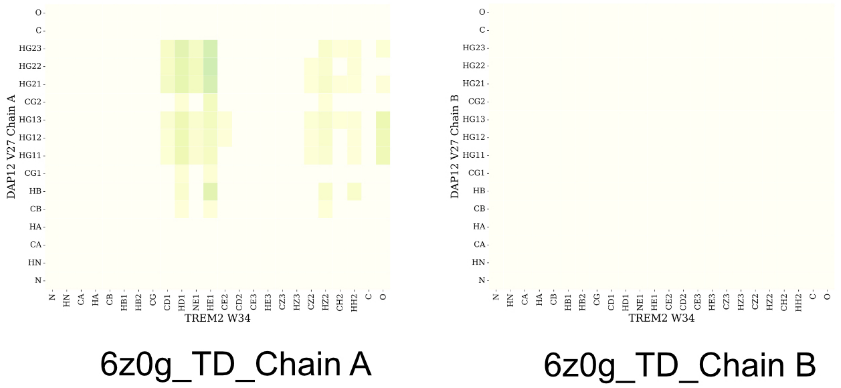

B

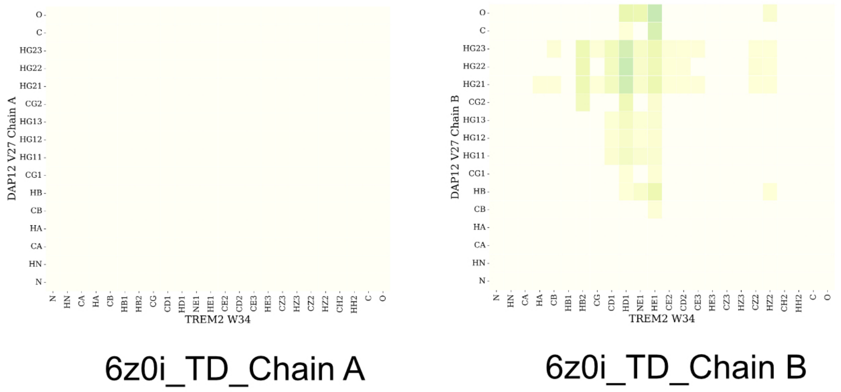

C

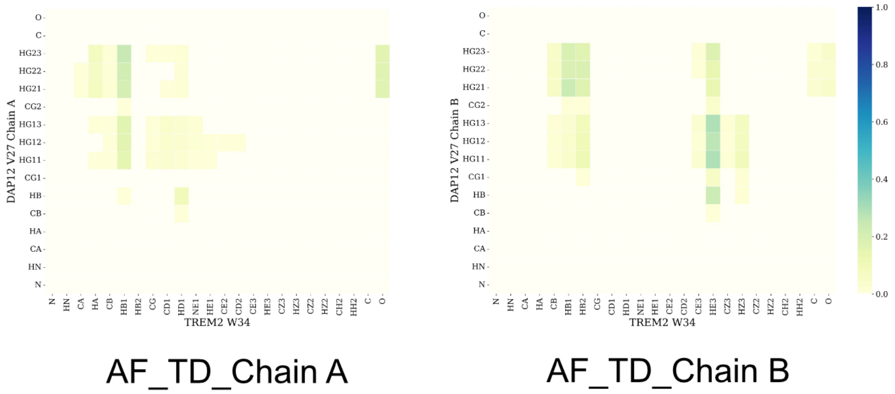

Figure S14: Contact maps of W34/V27 residues. (A) The contact maps of W34/V27 residues in 6z0g_TD system. (B) The contact maps of W34/V27 residues in 6z0i_TD system. (C) The contact maps of W34/V27 residues in AF_TD system.

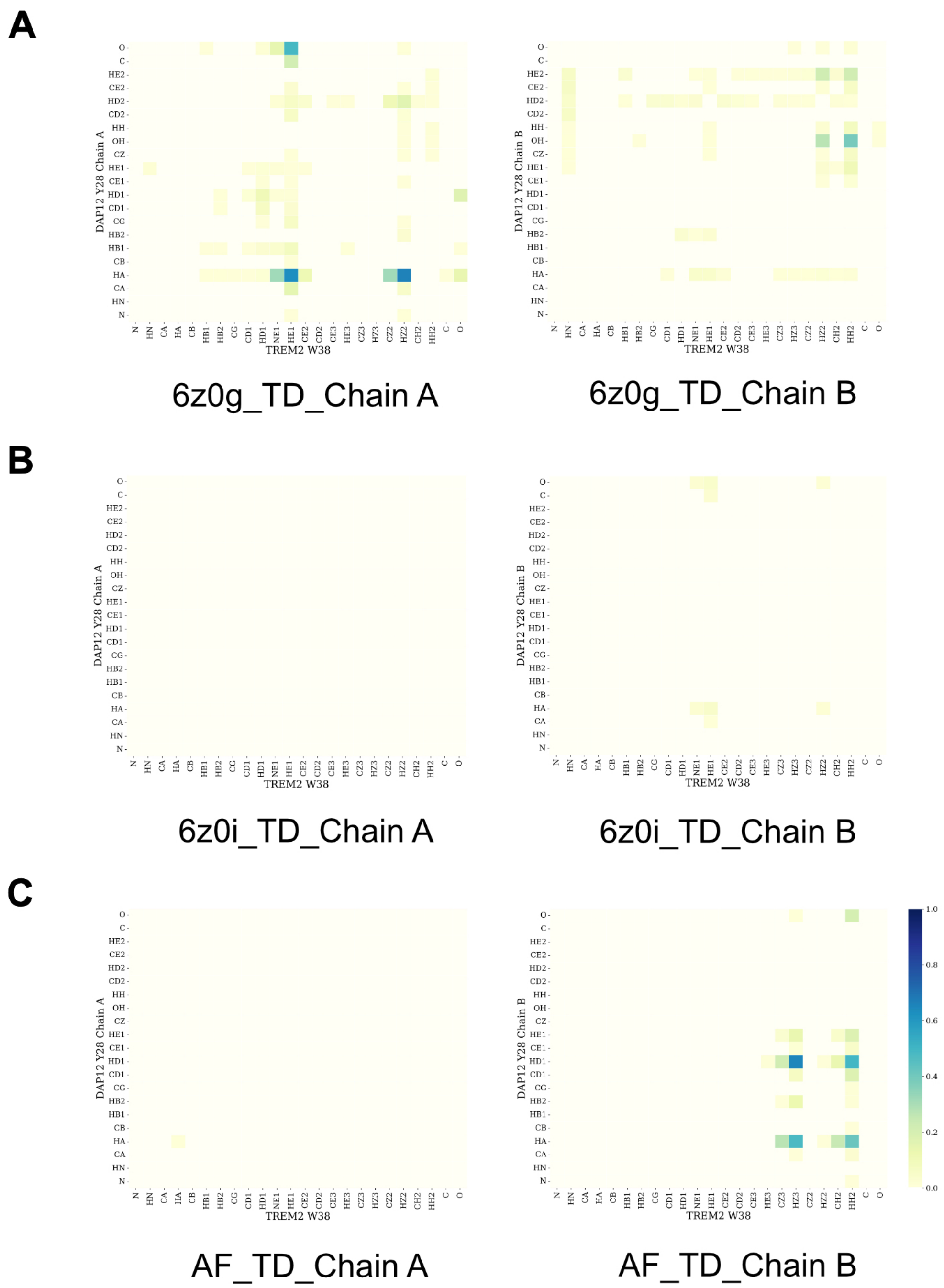

Figure S15: Contact maps of W34/Y28 residues. (A) The contact maps of W34/Y28 residues in 6z0g_TD system. (B) The contact maps of W34/Y28 residues in 6z0i_TD system. (C) The contact maps of W34/Y28 residues in AF_TD system.

Detailed hydrogen bond possibilities of the TREM2/DAP12

Table S1: Hydrogen bonds possibility of K26/D16.

system	HZ1-A[a]	HZ2-A	HZ3-A	Total-A	HZ1-B[a]	HZ2-B	HZ3-B	Total-B
6z0g_OD1	7.90	14.38	12.46	34.74	0	0	0	0
6z0g_OD2	20.20	9.52	14.85	44.57	0	0	0	0
6z0i_OD1	6.15	9.68	9.02	24.85	15.33	7.67	11.91	34.91
6z0i_OD2	14.77	16.90	28.68	60.35	19.83	11.23	11.97	43.03
AF_OD1	10.23	10.30	12.33	32.86	8.77	12.67	11.85	33.29
AF_OD2	13.33	11.89	13.27	38.49	11.95	12.85	9.98	34.78

[a]A is refered to Chain A. [b]B is refered to Chain B.

Table S2: Hydrogen bonds possibility of K26/T20.

system	HZ1-A	HZ2-A	HZ3-A	Total-A	HZ1-B	HZ2-B	HZ3-B	Total-B
6z0g_OG1	5.02	5.50	4.58	15.10	0.17	0.25	0.10	0.52
6z0i_OG1	0	0	0	0	0	0	0	0
AF_OG1	0	0	0	0	0.35	0.35	0.32	1.02

Table S3: Hydrogen bonds possibility of W34/T20.

system	HE1-A	HE1-B	system	HE1-A	HE1-B
6z0g_OG1	0	0	6z0g_O	0	0
6z0i_OG1	0	0	6z0i_O	0	0
AF_OG1	0.55	0	AF_O	9.08	0

Mergerd hydrogen bond interaction

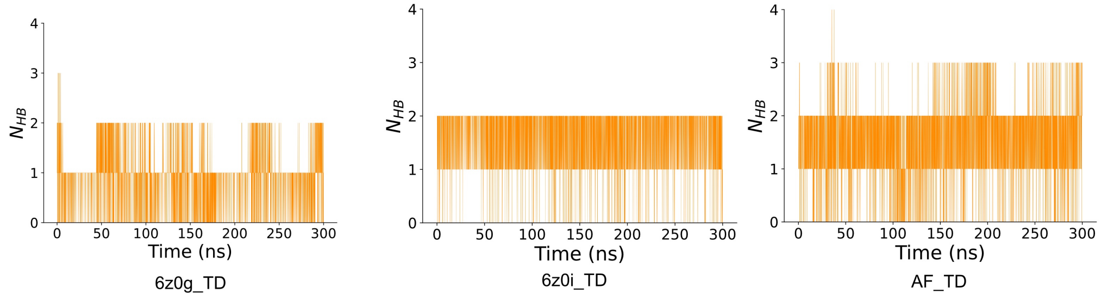

Figure S16: Residues distance of three systems.

Hydrophobic interaction of key residues

Figure S17, S18, S19, and S20 display the residue pairs involved in hydrophobic interactions, namely L19/I12, W23/I23, W34/A24, and W34/V27, respectively. Figure S21 illustrates the distance between these residue pairs to determine hydrophobic interactions. In this analysis, a hydrophobic interaction is defined when the distance between any carbon atoms within a particular residue and another residue is below 5 Å.

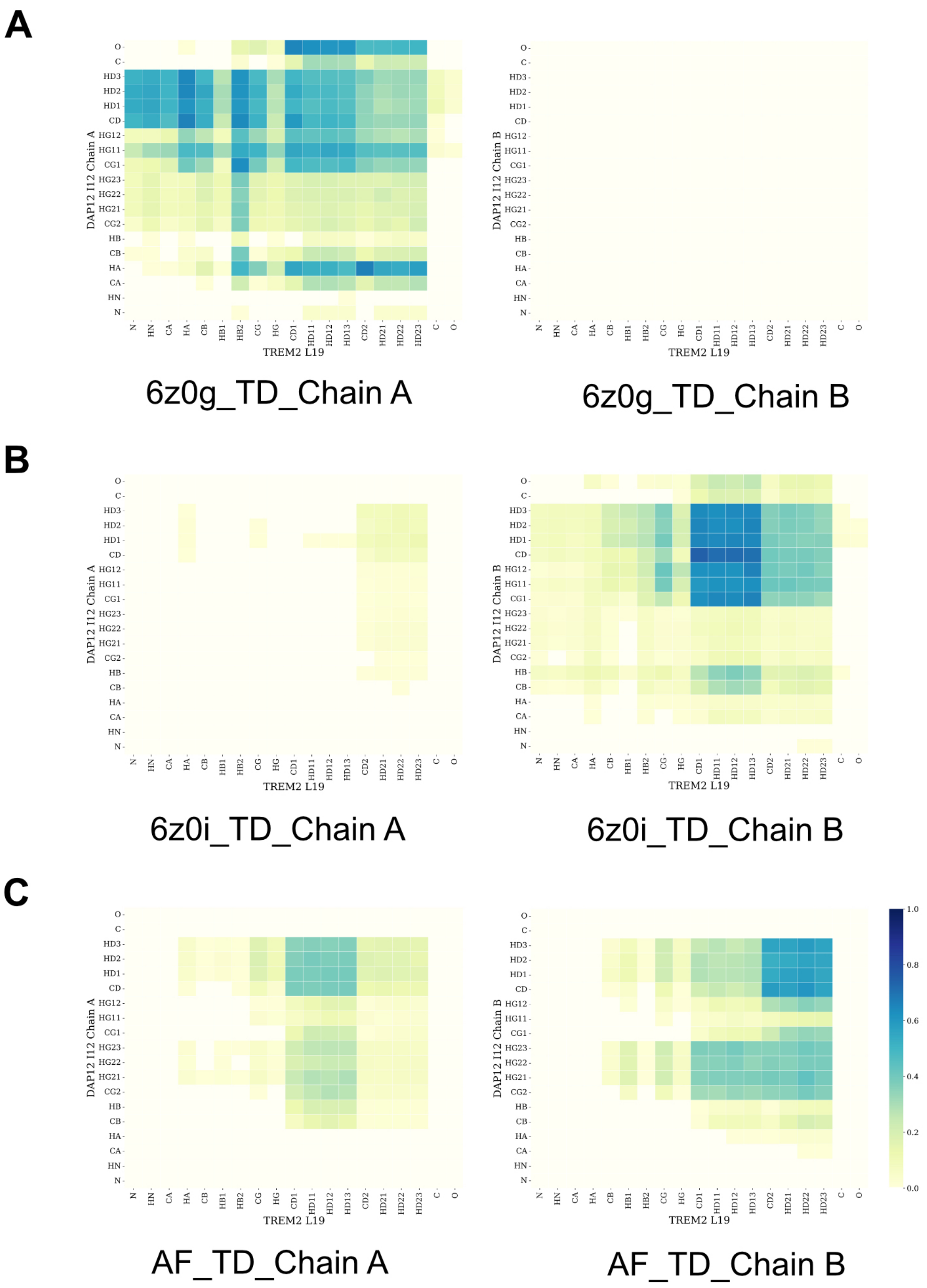

Figure S17: Hydrophobic interaction of L19/I12 in three systems. (A) The hydrophobic interaction of L19/I12 residues in 6z0g_TD system. (B) The hydrophobic interaction of L19/I12 residues in 6z0i_TD system. (C) The hydrophobic interaction of L19/I12 residues in AF_TD system.

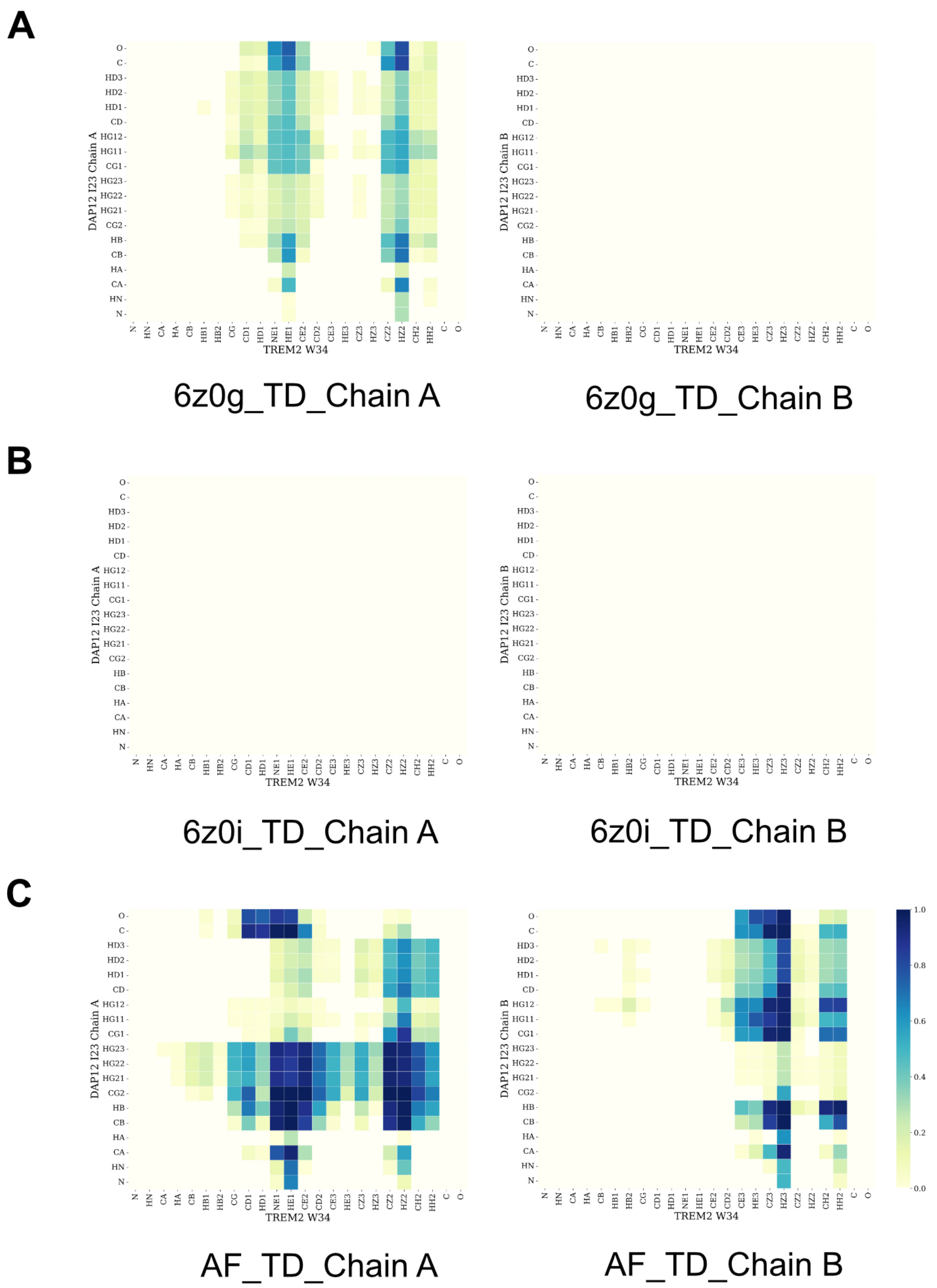

Figure S18: Hydrophobic interaction of W23/I23 in three systems. (A) The hydrophobic interaction of W23/I23 residues in 6z0g_TD system. (B) The hydrophobic interaction of W23/I23 residues in 6z0i_TD system. (C) The hydrophobic interaction of W23/I23 residues in AF_TD system.

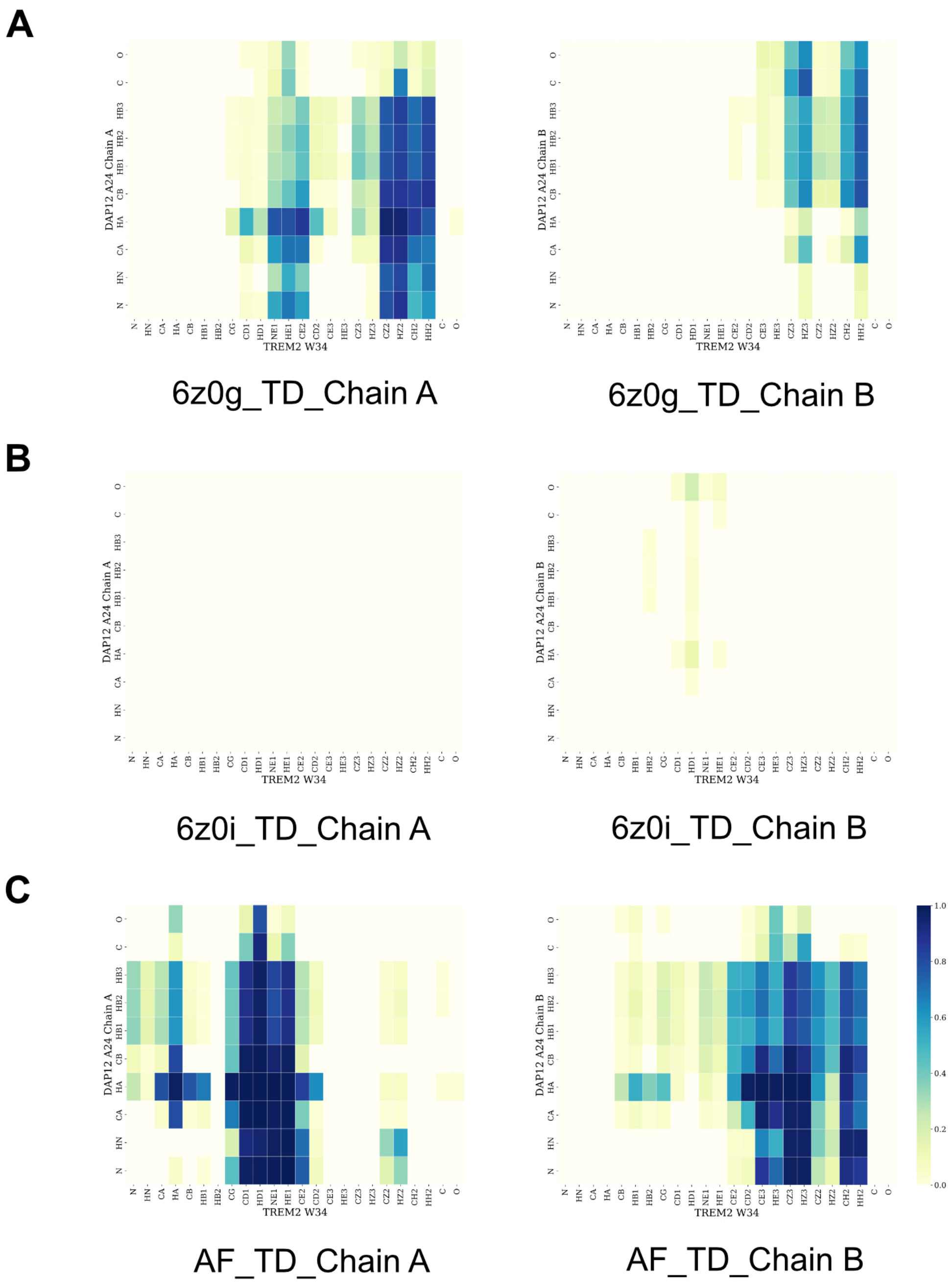

Figure S19: Hydrophobic interaction of W34/A24 in three systems. (A) The hydrophobic interaction of W34/A24 residues in 6z0g_TD system. (B) The hydrophobic interaction of W34/A24 residues in 6z0i_TD system. (C) The hydrophobic interaction of W34/A24 residues in AF_TD system.

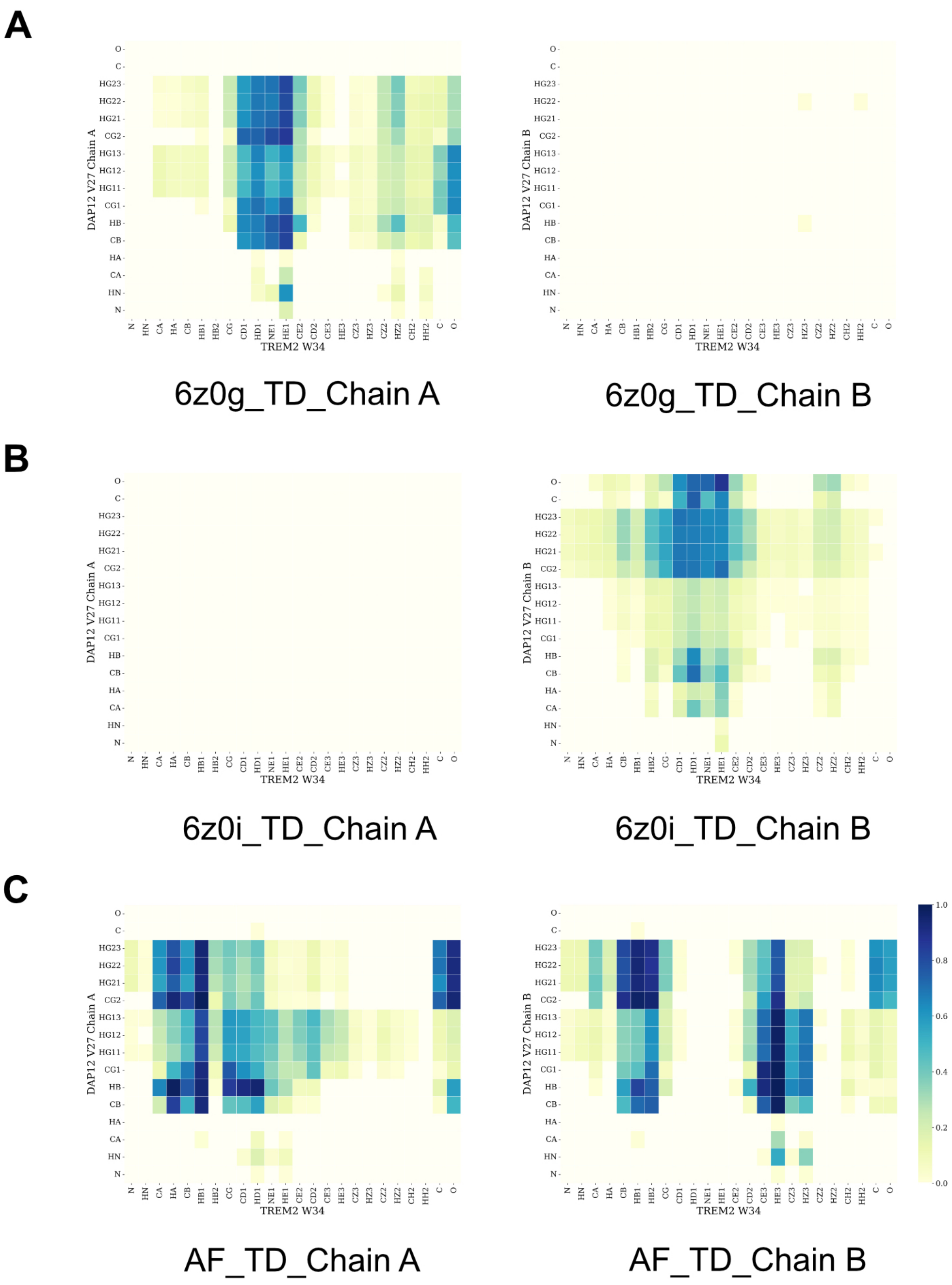

Figure S20: Hydrophobic interaction of W34/V27 in three systems. (A) The hydrophobic interaction of W34/V27 residues in 6z0g_TD system. (B) The hydrophobic interaction of W34/V27 residues in 6z0i_TD system. (C) The hydrophobic interaction of W34/V27 residues in AF_TD system.

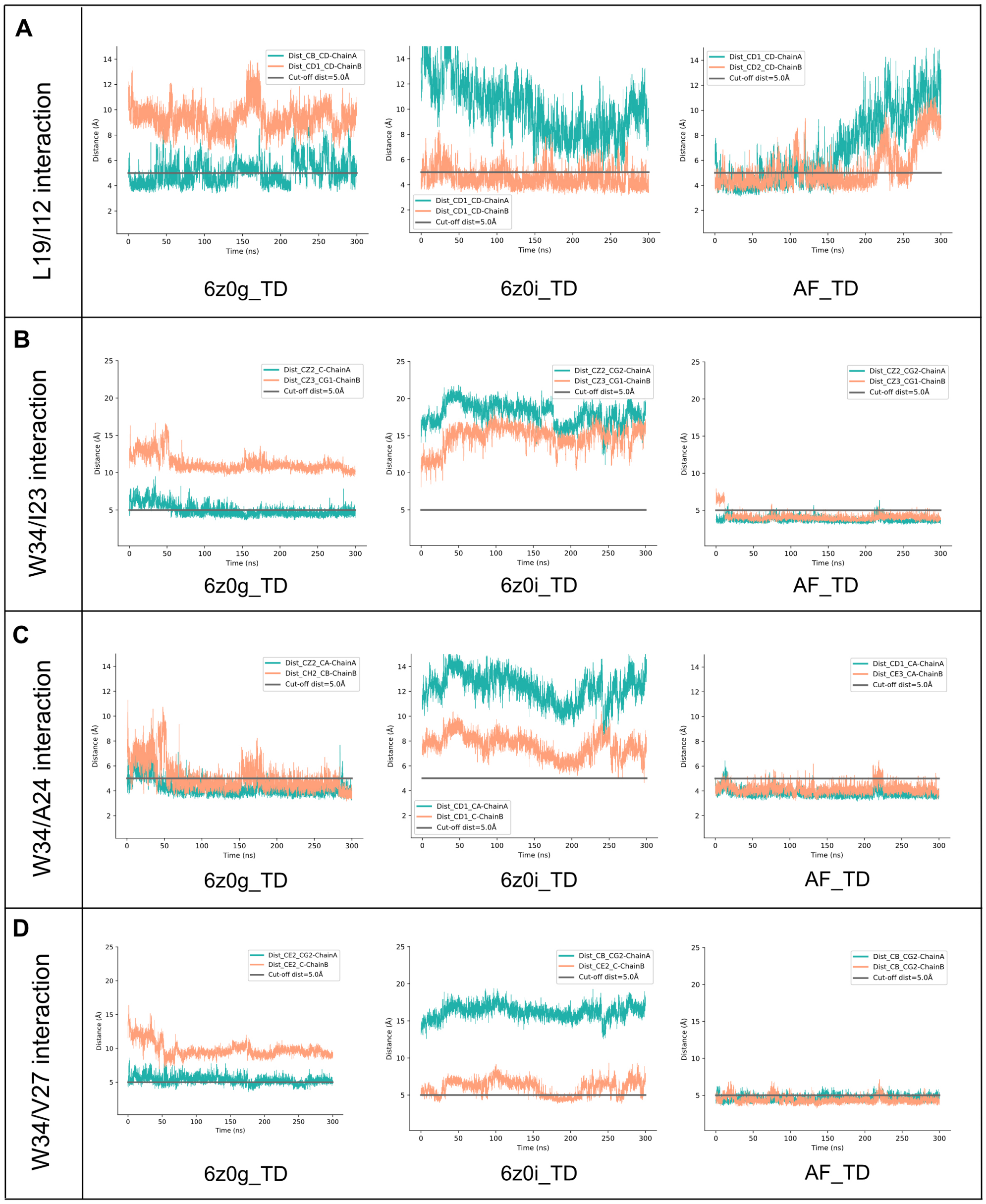

Figure S21: Hydrophobic interaction of key residues in three systems. (A) The hydrophobic interaction of L19/I12 residues in three systems. (B) The hydrophobic interaction of W34/I23 residues in three systems. (C) The hydrophobic interaction of W34/A24 residues in three systems. (D) The hydrophobic interaction of W34/V27 residues in three systems.

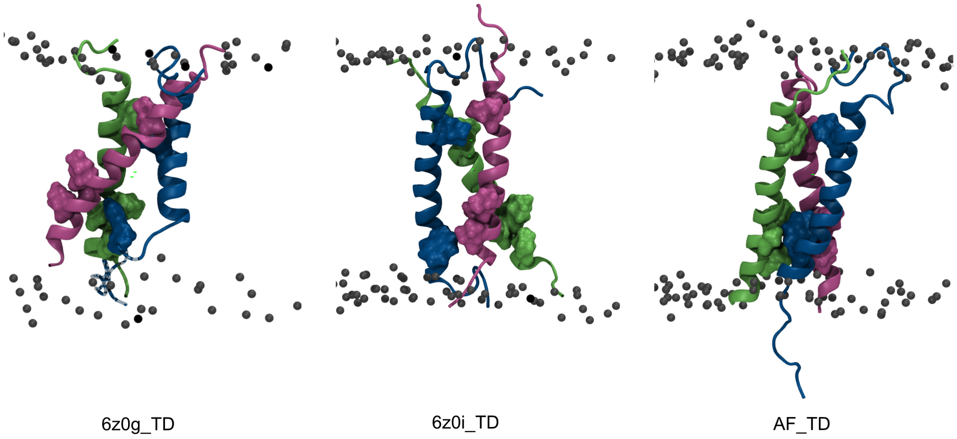

Figure S22: Hydrophobic residues facing direction in three systems.

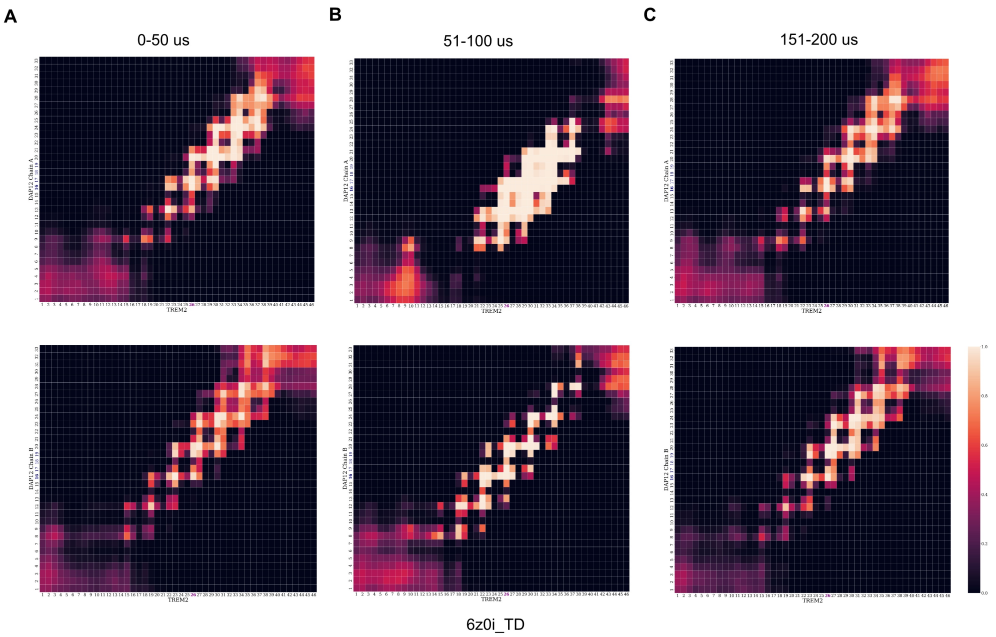

Figure S23: Contact maps of different time frames of CG simulation. (A) Contact maps of 6z0i_TD in 0-50 us. Top map showing the contacts of TREM2 and DAP12 chain A, and the bottom map showing the contacts of TREM2 and DAP12 chain B. (B) Contact maps of 6z0i_TD in 50-100 us. (C) Contact maps of 6z0i_TD in 150-200 us.

Table S4: Sequence of three systems.

**chains**	**sequence**
DAP12 Chain A	*CSTVSPGVLAGIVVGDLVLTVLIALAVYFLGRL*
DAP12 Chain B	*CSTVSPGVLAGIVVGDLVLTVLIALAVYFLGRL*
TREM2	*RSLLEGEIPFPPTSILLLLACIFLIKILAASALWAAAWHGQKPGTH*
